# STIM2 is involved in the regulation of apoptosis and the cell cycle in normal and malignant monocytic cells

**DOI:** 10.1002/1878-0261.13584

**Published:** 2024-01-17

**Authors:** Stefan Djordjevic, Raphaël Itzykson, Frédéric Hague, Delphine Lebon, Julien Legrand, Hakim Ouled‐Haddou, Guillaume Jedraszak, Juliette Harbonnier, Louison Collet, Etienne Paubelle, Jean‐Pierre Marolleau, Loïc Garçon, Thomas Boyer

**Affiliations:** ^1^ HEMATIM UR4666 Université Picardie Jules Verne Amiens France; ^2^ Département Hématologie et Immunologie Hôpital Saint‐Louis, Assistance Publique‐Hôpitaux de Paris France; ^3^ Génomes, Biologie Cellulaire et Thérapeutique U944, INSERM, CNRS Université Paris Cité France; ^4^ Laboratoire de Physiologie Cellulaire et Moléculaire UR4667 Université Picardie Jules Verne Amiens France; ^5^ Service d'Hématologie Clinique et de Thérapie Cellulaire CHU Amiens‐Picardie France; ^6^ Laboratoire de Génétique Constitutionnelle CHU Amiens‐Picardie France; ^7^ Service d'Hématologie Biologique CHU Amiens‐Picardie France

**Keywords:** apoptosis, calcium, genomic stress, leukemia, monocytic cells, SOCE

## Abstract

Calcium is a ubiquitous messenger that regulates a wide range of cellular functions, but its involvement in the pathophysiology of acute myeloid leukemia (AML) is not widely investigated. Here, we identified, from an analysis of The Cancer Genome Atlas and genotype‐tissue expression databases, stromal interaction molecule 2 (*STIM2*) as being highly expressed in AML with monocytic differentiation and negatively correlated with overall survival. This was confirmed on a validation cohort of 407 AML patients. We then investigated the role of STIM2 in cell proliferation, differentiation, and survival in two leukemic cell lines with monocytic potential and in normal hematopoietic stem cells. STIM2 expression increased at the RNA and protein levels upon monocyte differentiation. Phenotypically, *STIM2* knockdown drastically inhibited cell proliferation and induced genomic stress with DNA double‐strand breaks, as shown by increased levels of phosphorylate histone H2AXγ (p‐H2AXγ), followed by activation of the cellular tumor antigen p53 pathway, decreased expression of cell cycle regulators such as cyclin‐dependent kinase 1 (CDK1)–cyclin B1 and M‐phase inducer phosphatase 3 (CDC25c), and a decreased apoptosis threshold with a low antiapoptotic/proapoptotic protein ratio. Our study reports STIM2 as a new actor regulating genomic stability and p53 response in terms of cell cycle and apoptosis of human normal and malignant monocytic cells.

## Graphical abstract

This research uncovers an important role of STIM2 in the proliferation and survival of normal and malignant monocytic cells. STIM2 knockdown in both cell type leads to decreased cell proliferation and survival through induction of genotoxic stress and induction of p53, leading to cell cycle block and apoptosis in both cell types. STIM2 may be a potential target in AML treatment.

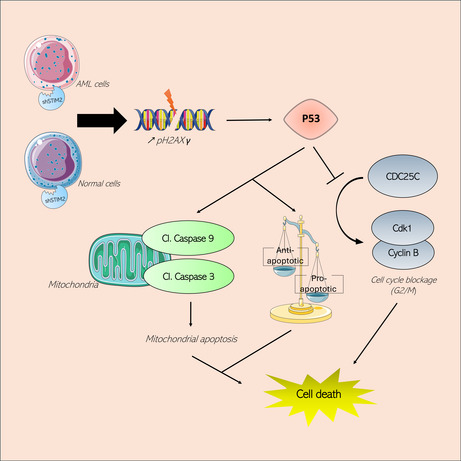

**Abbreviations**
AM: acetoxymethylester
AML: acute myeloid leukemia
Ca^2+^
: calcium
CaM: calmodulin
CAMK: Ca^2+^/calmodulin‐dependent protein kinase
CFU‐GM: granulocyte–macrophage colony‐forming units
CLARA: clofarabine–cytarabine
CR: complete remission
CRp: CR with incomplete platelet recovery
DAPI: 4′,6‐diamidino‐2‐phenylindole
DFS: disease‐free survival
ER: endoplasmic reticulum
FAB: French–American–British classification
FBS: fetal bovine serum
FLT3‐L: FMS‐like tyrosine kinase 3 ligand
GFP: green fluorescent protein
GM‐CSF: granulo‐macrophage colony‐stimulating factor
GTEx: genotype‐tissue expression
HSC: hematopoietic stem cells
IL3: interleukin 3
ITPR: inositol 1,4,5‐triphosphate receptor
KD: knockdown
M‐CSF: macrophage colony‐stimulating factor
NFAT: nuclear factor of activated T cells
OS: overall survival
PFT‐α: pifithrin‐α
PH: proportional hazard
PKC: protein kinase C
RT‐qPCR: quantitative reverse transcriptase‐polymerase chain reaction
shRNA: short hairpin RNA
shSCR: shSCRAMBLE
siRNA: small interferent RNA
SOCE: store‐operated calcium entry
TCGA: The Cancer Genome Atlas
TPO: thrombopoietin
TRPM: transient receptor potential melastatin
WBC: white blood cell

## 1 Introduction

Acute myeloid leukemia (AML) are hematological neoplasms characterized by overproduction of oligoclonal progenitor cells, maturation arrest, and subsequent accumulation of blast cells at various stages of incomplete differentiation in bone marrow [1]. AML present in genetic, epigenetic, and subclonal heterogeneity, which leads to significant phenotypic variability causing reduced therapeutic efficacy [2, 3]. The prognosis of AML remains poor with a standardized net survival estimated at 50% at 1 year and 27% at 5 years; this prognosis worsens with the age of the patients [4]. Novel targeted therapies are emerging, some being now part of the standard of care of patients, but they still have limits of effectiveness. It is therefore required to better understand the mechanisms of leukemogenesis and identify new pathophysiological processes that could be targeted in the future.

Calcium ions (Ca^2+^) are second messengers in numerous cell signaling pathways, contributing to physiological responses such as proliferation, differentiation, and apoptosis [5, 6, 7, 8]. At steady state, the intracellular Ca^2+^ concentration in the cytosol is as low as 10 nm, but increases due to an influx from the extracellular compartment through channels expressed at the plasma membrane that respond to various stimuli such as depolarization, stretch, noxious stimuli, and extracellular agonists [9]. Increased cytosolic Ca^2+^ concentration can also rise from intracellular stores, such as the endoplasmic reticulum (ER) and mitochondria [10]. One of the most important triggers of Ca^2+^ influx is the store‐operated calcium entry (SOCE), a ubiquitous pathway controlled by the intracellular ER Ca^2+^ store [11, 12]. Indeed, its depletion activates 2 proteins located at the ER membrane, STIM1 and STIM2 [13, 14], which form oligomers and translocate to the plasma membrane. This activates proteins such as ORAI to form a functional channel that allows Ca^2+^ entry from the extracellular medium [15, 16]. Many reports have highlighted the role of Ca^+^ actors in general and SOCE in particular in solid tumors. As an example, a high ORAI1 expression is observed in breast [17], ovary carcinoma [18], esophagus [19], kidney [20], and many other malignant tumors [21, 22], while an increased ORAI3 expression is associated with a worsened prognosis in breast [23, 24] and prostate cancer [25].

Surprisingly, the abundant literature concerning the involvement of Ca^2+^ modulators in carcinogenesis contrasts with the lack of data in hematological malignancies. However, Ca^2+^ regulates normal hematopoiesis at different levels. In hematopoietic stem cells (HSC), it controls the balance between quiescence and proliferation, and is involved in cell interaction within the bone marrow niche [26]. At later stage, it is involved in cell commitment and proliferation through activation of Ca^2+^‐related signaling pathways such as the calcium/calmodulin‐dependent (CaM)/CaM kinase kinase/CaM kinase axis, NFAT, phospholipase C and protein kinase C [27]. At the progenitor level, IL3 or GM‐CSF as well as the P2 receptor, increase Ca^2+^ concentration, and thereby control the proliferation/differentiation balance depending on the amplitude of Ca^2+^ entry [27]. Considering the growing evidence on Ca^2+^ function in the control of normal hematopoiesis, it is reasonable to think that its deregulation would be involved in leukemogenesis. The Ca^2+^/CaM complex regulates cell cycle progression and phosphorylation of Rb protein in the leukemic cells HL60 [28]. Activation of CAMKIIγ inhibits their differentiation, which occurs after exposure to KN62, a CAMKII inhibitor. A high level of CAMKIIγ phosphorylation in primary AML blasts and AML cell lines favors cell proliferation at the expense of differentiation [29]. Abnormal expression of Ca^2+^ channels has also been described in AML. TRPM2 is highly expressed in AML and its knockdown in U937 cells decreases proliferation and increases sensitivity to doxorubicin [30]. Expression of ITPR2, which regulates mobilization of Ca^2+^ from its storage, is high in AML and correlates with a worsened prognosis [31]. Focusing on SOCE actors, ORAI2 expression is high in HL60 cells, and its knockdown decreases cell proliferation [32]. In parallel, a recent report showed chemoresistance for AML cells treated with cytarabine with elevated ORAI1 expression that confirms the involvement of SOCE in AML cell lines and primary AML blasts, controlling both cell cycle and proliferation in KG1a and U937 cell lines [33].

In order to identify Ca^2+^ actors that may be involved in malignant myelopoiesis, we performed a systematic analysis of overall survival (OS) according to the expression of selected candidate proteins in AML patients from the Cancer Genome Atlas (TCGA) and genotype‐tissue expression (GTEx) databases and in a cohort of 407 AML patients included in the ALFA 0702 protocol. We identified STIM2 as heterogeneously expressed in AML, with a negative prognostic value when highly expressed in terms of OS. STIM2 is an ER‐resident protein that regulates Ca^2+^ concentration via SOCE. It has been proposed to be an important player in age‐related pathologies, Alzheimer's [34] and Huntington's disease [35], autoimmune diseases, and cancers [36]. In addition, STIM2 has been shown to be significantly involved in mice [37], particularly in the nervous [38] and the immune system in cooperation with STIM1 [39]. Despite the growing evidence of STIM2 function, very little is known on its involvement in myeloid malignancies and during normal hematopoiesis. Using shRNA‐based STIM2 knockdown in two myeloid leukemic cell lines, THP1 and OCI‐AML3, and in primary CD34^+^ cells driven into *in vitro* myeloid differentiation, we describe here the role of STIM2 in proliferation, cell cycle control, and survival of monocytic cells.

## 2 Materials and methods

### 2.1 Cell lines culture and reagents

The human AML cell line OCI‐AML3 (RRID: CVCL_1844, AML FAB M4) was ordered from DMSZ and cultured in alpha‐MEM (Sigma‐Aldrich, Burlington, MA, USA) supplemented with 2 mm l‐glutamine (Eurobio, Les Ulis, France), containing 10% FBS (PAN Biotech, Aidenbach, Germany), 100 U·mL^−1^ penicillin (Eurobio) and 100 μg·mL^−1^ streptomycin (Eurobio). THP1 cells (RRID: CVCL_0006, AML FAB M5) were ordered from ATCC (ATCC, Manassas, VA, USA) and cultured in RPMI‐1640 (Biosera, Cholet, France) medium supplemented with 10% FBS, 100 U·mL^−1^ penicillin, and 100 μg·mL^−1^ streptomycin. These cell lines were authenticated in the past 3 years using flow cytometry according to the product sheet. Absence of Mycoplasma was assessed every other week using a bioluminescence‐based assay (Mycoplasma Detection Kit‐QuickTest; Biotool, Houston, TX, USA).

For differentiation into monocyte‐like cells, THP1 and OCI‐AML3 cells were treated with 1 μm 1,25(OH)_2_D_3_ (Calbiochem, Millipore, Burlington, MA, USA) for 72 h. To suppress the function of p53 protein, α‐pifithrin (PFT‐α) (TargetMol, Wellesley Hills, MA, USA) was used at 10 μm in the medium.

### 2.2 *In vitro* culture of human CD34^+^ progenitors

CD34^+^ progenitor cells were sorted by magnetic microbead separation on MACS columns (AutoMACS; Miltenyi Biotec, Bergisch Gladbach, Germany) from cytapheresis samples from healthy donors. *In vitro* monocytic differentiation was driven in IMDM‐based (Biochrom, Merck‐Millipore, Burlington, MA, USA) containing 15% FBS (PAN Biotech), 100 U·mL^−1^ penicillin (Eurobio), 100 μg·mL^−1^ streptomycin (Eurobio), recombinant human interleukin‐3 (rhIL‐3 10 ng·mL^−1^; Miltenyi, Bergisch Gladbach, Germany), macrophage colony‐stimulating factor (M‐CSF 50 ng·mL^−1^; Miltenyi), thrombopoietin (TPO 20 ng·mL^−1^; Miltenyi), and human FMS‐like tyrosine kinase 3 ligand (FLT3‐L 50 ng·mL^−1^; Miltenyi) from Days 0 to 21. Monocytic differentiation was monitored using flow cytometry every 2–3 days.

### 2.3 Clonogenic potential

Transduced CD34^+^ cells were sorted according to the GFP expression and then plated in triplicate at a density of 300 cells in 1 mL of semisolid MethoCult H4435‐enriched medium (Stem Cell Technologies, Vancouver, BC, Canada) and incubated for 14 days at 37 °C in a humidified atmosphere with 5% CO_2_. The granulocyte–macrophage colony‐forming units (CFU‐GM) were identified *in situ* with an inverted microscope (Nikon Eclipse TS100, Nikon, Tokyo, Japan).

### 2.4 Flow cytometry

Cells (5 × 10^4^) were stained with panels of conjugated antibodies in 200 μL of 1× phosphate‐buffered saline (PBS) listed in Table S1 4′,6‐diamidino‐2‐phenylindole (DAPI) staining was used to gate live cells. For cell cycle analysis, 10^6^ cells were centrifuged at 300 ***g*** for 5 min, and resuspended and incubated in 100 μL 4% paraformaldehyde (Thermo Fisher Scientific, Waltham, MA, USA) for 20 min at 4 °C. After one wash, cells were resuspended in 100 μL 1× permwash (BD, Franklin Lakes, NJ, USA) and Hoechst (1/10 000e) (Thermo Fisher) for 1 h in the dark at room temperature. Cells were then washed in 1× PBS and resuspended in 200 μL 1× PBS. Acquisitions were performed on MACSQuant flow cytometer, and data analysis was performed using Flowjo (v10; TreeStar Inc., San Carlos, CA, USA).

### 2.5 Lentiviral particle production and cell transduction

Two shRNAs against *STIM2* and one control scramble shRNA cloned in pLKO.1‐CMV‐tGFP vector were selected using the Mission shRNA tool and purchased from Sigma‐Aldrich (detailed sequences are provided in Table S2). Human STIM2 cDNA cloned in pLV[Exp]‐EGFP:T2A:Puro‐EF1A lentiviral vector was purchased from VectorBuilder (Chicago, IL, USA) (detailed sequences are provided in Table S3). Viral production was ensured in the HEK293T cell line (RRID: CVCL_0063), after transfection using Lipofectamine LTX with plus reagent (Thermo Fisher Scientific, Waltham, MA, USA) in antibiotic‐free high‐glucose Dulbecco Modified Eagle Medium (Dominique Dutscher, Bernolsheim, France). Lentiviral supernatant was harvested from Days 2 to 4 and filtered through a 0.45 μm polyvinylidene fluoride membrane (Millex‐HV 0.45 μm 33 mm; Merck‐Millipore), before ultracentrifugation 100 000 ***g*** for 90 min at 4 °C (Optima Beckman‐Coulter, Brea, CA, USA). Infection was performed overnight with 8 mg·mL^−1^ polybrene (Sigma‐Aldrich). In THP1 and OCI‐AML3 cells, we used an MOI 10 to infect 5 × 10^5^, both with shSCRAMBLE and shSTIM2.

### 2.6 Small interferent RNA (siRNA)

Cells were transfected using Lipofectamine LTX and Plus reagent (Thermo Fisher) in OptiMEM medium (Dominique Dutsher) according to the manufacturer's protocol and the study by Carralot et al. [40]. Between 100 000 and 200 000 cells are transfected with two different siRNAs targeting STIM2 (Table S4) and a control siRNA (siCONTROL) (Eurogentec, Seraing, Belgium).

### 2.7 Calcium signaling

Calcium imaging allows calcium concentration to be determined in real time. It was performed on the THP1 cell line. Changes in intracellular calcium concentration were measured using the fluorescent probe Fura‐2 charged with an AM (acetoxymethylester) group, enabling it to cross the cell plasma membrane. Upon entry, the AM group is hydrolyzed by esterases, producing a fluorescent signal in the cell cytoplasm. Fura‐2 is excited simultaneously at two wavelengths (340 and 380 nm), with emission at 510 nm. The binding of calcium to the probe results in a variation in fluorescence. Thus, fluorescence measured at 340 nm increases, whereas fluorescence at 380 nm decreases. 200 000 THP1 cells were transfected by lipofection for 24 h and 35 mm coverslip were pretreated with poly‐l‐lysine (8 mg·mL^−1^) for 24 h. The transfected cells were then seeded on the treated coverslip for the next 24 h in their culture medium. Forty‐eight hours after transfection, the Fura‐2AM probe was loaded at a final concentration of 3.3 μm for 45 min. A camera captured variations in Fura‐2 emission fluorescence. Throughout the experiment, a perfusion/aspiration system was used to control the extracellular environment. For quantification of SOC entry, THP1 cells were first perfused with an extracellular medium containing 2 mm calcium for 2 min. This medium was then replaced by a calcium‐free extracellular medium and supplemented with 1 μm Thapsigargin for 11 min. The cells were then perfused again with an extracellular medium containing 2 mm calcium for 5 min. Finally, cells were perfused in a calcium‐free solution for the last 5 min. To quantify SOC entry, we decided to use the classical quantification: Δ*F*/*F*_0_ expressed in percentage: ((peak of fluorescence − basal fluorescence)/(basal fluorescence)) × 100.

### 2.8 Quantitative reverse transcriptase‐polymerase chain reaction (RT‐qPCR)

RNA isolation was performed with a mini or microRNA (Qiagen© kit; Qiagen, Hilden, Germany). RNA quantity was determined with the NanodDrop ND‐100 Spectrophotometer (Thermo Fisher Scientific). For each sample, 500 ng of RNA was used for reverse transcription reaction (Reverse Transcription Kit; Thermo Fisher). Gene expression was assessed by RT‐qPCR, using SYBR^®^ Green on QuantStudio7 device (Applied Biosystem, Waltham, MA, USA). The comparative *C*_T_ method was used for quantification of gene expression, and relative expression levels were calculated with a normalization to *GAPDH* or *HPRT* expression. Primer sequences are listed in Table S5.

### 2.9 Western blot analysis

A minimum of 200 000 cells were washed in 1× PBS and lysed on ice in RIPA buffer (Sigma‐Aldrich), containing protease and phosphatase inhibitors (Thermo Fisher). Before centrifugation (25 min, 13 000 ***g*** at 4 °C), the lysates were gently sonicated and the protein concentration was quantified using Bradford assay (Interchim, San Diego, CA, USA). After transfer and block in 5% of no‐fat milk, membranes were incubated overnight at 4 °C with the primary antibody solution 1/1000 diluted (detailed antibodies in Table S6). Bound primary antibodies were detected using anti‐mouse (Sigma‐Aldrich) or anti‐rabbit (Thermo Fisher) horseradish peroxidase‐conjugated secondary antibodies after a 1 h incubation at room temperature. Membranes were then exposed to the chemoluminescent substrate (Thermo Fisher), and the signal was detected using Chemidoc Device (Bio‐Rad, Hercules, CA, USA).

### 2.10 Nanostring nCounter assay

RNAs extracted from THP1 with or without STIM2 knockdown were assessed using the Nanostring PanCancer pathway panel according to the manufacturer's protocol. The protocol was performed over 48 h and the ‘processing’ lasts approximately 6 h. Data are analyzed on the nsolver™ Software v 4.0 (Nanostring Technologies, Seattle, WA, USA). The Plexset study was performed on samples obtained from the ALFA 0702 protocol (*n* = 407) in collaboration with the genomics platform and the pathology biology center with the agreement of the ALFA group's scientific advisory board. ELN 2017 risk stratification [41] was done as published [42]. Median follow‐up was 4.1 years (95% Cl 3.9–4.3 years). STIM2 expression and white blood cell (WBC) count were analyzed as Log_10_‐transformed continuous variables. STIM2 expression was dichotomized at median expression value. Univariable analyses were performed with Mann–Whitney or Kruskal–Wallis tests for dichotomic and other categorical variables, respectively. Multivariable analyses were done with logistic regressions. Censored data were estimated according to the Kaplan–Meier method [43]. Complete remission (CR) or CR with incomplete platelet recovery (CRp) OS and disease‐free survival (DFS) were defined according to ELN recommendations [41]. Univariable and multivariable analyses were done using log‐rank tests and Cox proportional hazard (PH) models, respectively [44]. In the latter, the PH assumption was estimated by graphical inspection of Schoenfeld residuals. Cumulative incidence of relapse (CIR) and No‐relapse mortality (NRM) were analyzed as competing risk with Fine & Grey models [45]. All multivariable analyses accounted for the ALFA0702 randomization arm (clofarabine–cytarabine [CLARA] versus cytarabine alone). All analyses were conducted in r 4.2.3 (RStudio, Boston, MA, USA).

### 2.11 Gene expression analysis database

The analysis of gene expression of the main gene of this study STIM2 was performed using the GEPIA2 site and tool (http://gepia2.cancer‐pku.cn/#index) [46]. This site allows a study of the expression of the gene of interest by compiling the data of the LAML cohort from the TCGA and GTEx. The use of this tool allows to study the expression profile of tumor versus nontumor samples and compare them.

### 2.12 Statistical analysis

For quantitative variables, we used Student's *t‐*test or one‐way analysis of variance and Tukey's *post hoc* analysis for multiparametric analysis. All numeric values are presented as the mean values ± standard error of the mean (SEM). All statistical tests were two‐tailed and the significance level was set to 0.05. Statistical analysis was performed with graphpad prism 8 software (GraphPad Prism Software Inc., San Diego, CA, USA).

## 3 Results

### 3.1 STIM2 expression pattern and prognosis value in AML samples

**Fig. 1:**
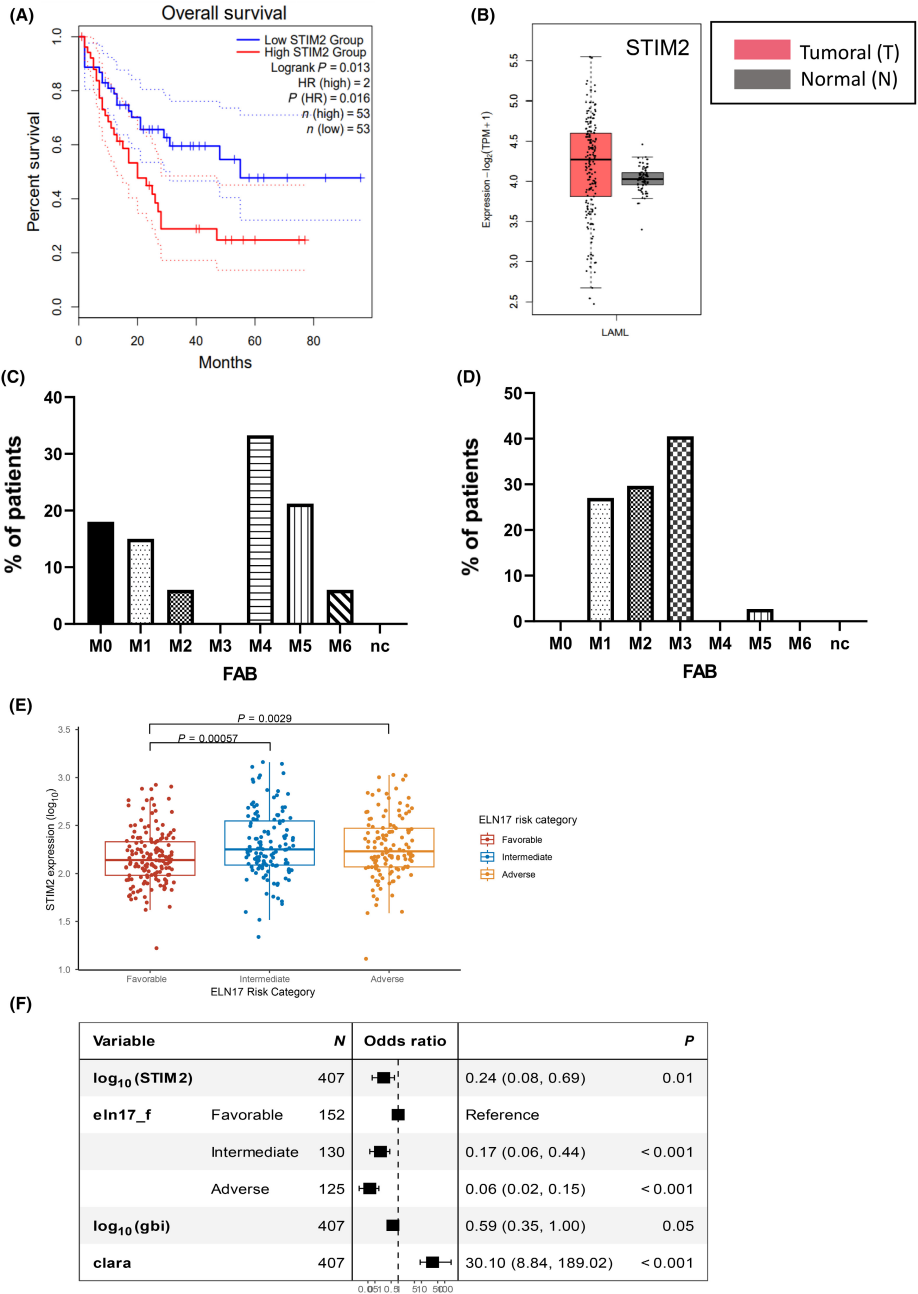
STIM2 expression pattern and prognosis value in acute myeloid leukemia samples. (A, B) Survival heatmaps were generated using GEPIA2 with the Cancer Genome Atlas data for Kaplan–Meier curves for (A) overall survival and for (B) whisker boxplots of the relative mRNA expression of STIM2 datasets. Survival is expressed as hazard ratio (HR). (C, D) Analysis of STIM2 expression depending on French–American–British (FAB) subtypes. The 50% of patients in the upper quartile (C) and the 50% of patients in the lower quartile (D). (E) STIM2 expression across European LeukemiaNet 2017 (ELN 2017) risk groups (*n* = 407), significant differences were assessed using the Mann–Whitney *U*‐test. (F) Lower odds of reaching complete remission of higher STIM2 expression expressed as a continuous variable (*n* = 407). Vertical dashed line indicates an odds ratio of 1. Error bars represent 95% confidence intervals. Multivariable analyses were done with logistic regressions. AML, acute myeloid leukemia; Clara, clofarabine–cytarabine; M0, undifferentiated acute myeloblastic leukemia; M1, acute myeloblastic leukemia with minimal maturation; M2, acute myeloblastic leukemia with maturation; M3, acute promyelocytic leukemia; M4, acute myelomonocytic leukemia; M5, acute monocytic leukemia; M6, acute erythroid leukemia.

To identify Ca^2+^ actors with prognosis value in AML, we performed a comparative analysis of OS according to the expression of several candidates in AML patients from the TCGA and GTEx databases, using GEPIA2 site and tool. Among them, we selected STIM2 for further studies, since its expression, although heterogeneous in AML, was statistically correlated with a worsened OS (Fig. 1A,B). We performed a second analysis on TCGA database that included 200 AML patients for whom FAB classification was available. We observed that AML with the highest STIM2 expression presented frequent monocytic or myelomonocytic differentiation (Fig. 1C), whereas this pattern of differentiation was absent in AML with the lowest STIM2 expression (Fig. 1D). We then investigated STIM2 expression using the nCounter^®^ PlexSet™ technology in a cohort of 407 patients included in the ALFA 0702 protocol with evaluable ELN17 risk [47]. As a continuous variable, STIM2 expression differed across ELN 2017 risk groups (Kruskal, *P* = 0.0007). Specifically, compared with Fav risk AML, both intermediate (Mann–Whitney, *P* = 0.00057) and adv (*P* = 0.0029) AMLs had higher STIM2 expression (Fig. 1E). In univariable analysis, there was a nonsignificant trend for lower STIM2 expression in patients achieving CR/CRp (*n* = 346) compared with those failing (Fig. S1A). In a multivariable logistic regression, the lower odds of reaching CR of higher STIM2 expression were significant both as a continuous variable (OR = 0.24, 95% CI 0.08–0.69, *P* = 0.01 for a 10‐fold increase in expression) (Fig. 1F) or as a dichotomic variable (OR = 0.50, 95% CI 0.25–0.97, *P* = 0.04) (Fig. S1B) independent of ELN risk, WBC count and ALFA0702 randomization arm. Though STIM2 expression either as a continuous or dichotomic variable, had no impact on relapse incidence or nonrelapse death after CR (data not shown), there was a nonsignificant trend for shorter OS in patients with higher STIM2 expression as a continuous variable (HR = 1.59, 95% CI 0.93–2.73, *P* = 0.09) when accounting for ELN risk, WBC count and randomization arm (data not shown).

### 3.2 STIM2 knockdown alters SOCE and decreases cell proliferation in THP1 and OCI‐AML3 cells

**Fig. 2:**
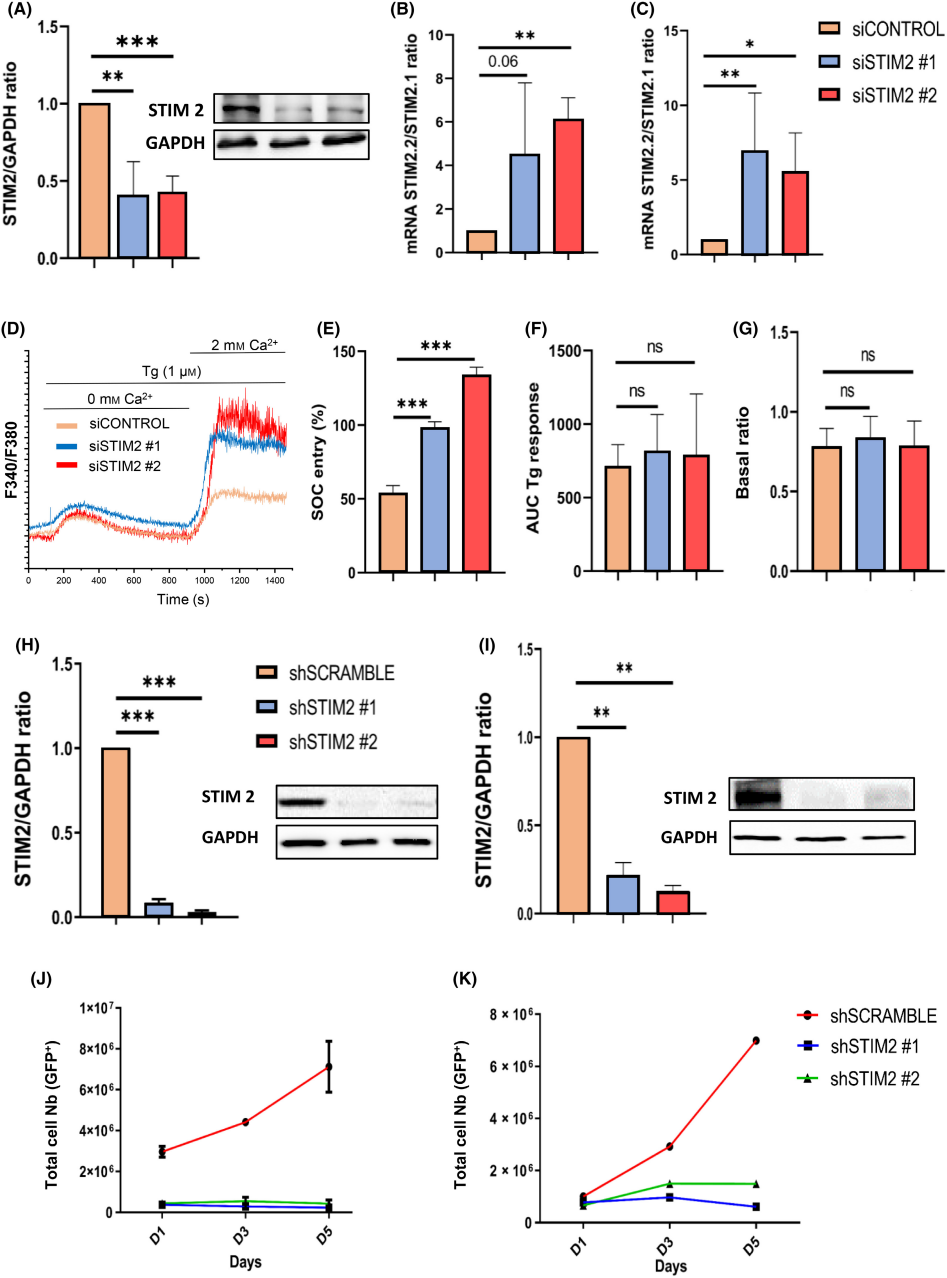
STIM2 knockdown alters store‐operated calcium entry (SOCE) and decreases cell proliferation in THP1 and OCI‐AML3. All data were obtained (western blot, calcium imaging, isoform, RT‐qPCR, and cell count) at 48 h postinfection for siRNA and 72 h for shRNA. (A) In THP1 protein expression of STIM2 cells (si#1: 0.407 ± 0.219; si#2: 0.410 ± 0.123) as determined by western blot relative to GAPDH (*n* = 3). (B, C) The STIM2.2/STIM2.1 ratio was performed by quantitative PCR in THP1 (si#1: 4.54 ± 3.259; si#2: 6.120 ± 0.9966) (B) (*n* = 4) and in OCI‐AML3 (sh#1: 6.98 ± 3.84; sh#2: 5.60 ± 2.55) (C) (*n* = 5). (D) Representative trace of SOCE measured with the ratio F340/F380 in THP1 cells after STIM2 silencing (*n* = 4) (siCONTROL *n* = 57; si#1 *n* = 72; si#2 *n* = 90). Cells were exposed to 1 μm Thapsigargin in the absence of Ca^2+^ which depletes the intracellular calcium (Ca^2+^). Extracellular Ca^2+^ concentration was then brought to 2 mm to induce SOCE. (E–G) Quantification of SOCE (siCONTROL: 53.63 ± 49.61; si#1: 93.33 ± 51.78; si#2: 134.2 ± 53.02) (*n* = 6) (siCONTROL *n* = 87; si#1 *n* = 175; si#2 *n* = 112) (E), Thapsgiargin (TG)‐response (siCONTROL: 744 ± 142; si#1: 816 ± 229; si#2: 740 ± 257) (*n* = 4) (F) and basal calcium (siCONTROL: 0.808 ± 0.108; si#1: 0.890 ± 0.169; si#2: 0.811 ± 0.123) (*n* = 4) (G). (H) STIM2 protein expression in THP‐1 cells (sh#1: 0.08 ± 0.02; sh#2: 0.03 ± 0.01) was determined by western blot relative to GAPDH (*n* = 3). (I) STIM2 protein expression in OCI‐AML3 cells (sh#1: 0.220 ± 0.0693; sh#2: 0.130 ± 0.03) was determined by western blot relative to GAPDH (*n* = 3). (J, K) In THP1 cells, proliferation was assessed by trypan blue cell count and then reported to the numbers (Nb) of GFP cells at each cytometry point (Days 1 – 3 – 5 (D1 – 3 – 5)) (*n* = 3) (J) and in OCI‐AML3 (*n* = 3) (K). All numeric values are presented as the mean values ± standard error of the mean. *P*‐values are calculated using one‐way ANOVA, ***P < 0.001; **P < 0.01; *P < 0.05; NS, not significant.

We assessed STIM2 expression in two models of leukemic cell lines, THP1 and OCI‐AML3, that can be driven *in vitro* toward monocytic differentiation by vitamin D (Fig. S2). In both cell lines, STIM2 expression increased significantly at the RNA and protein levels after vitamin D exposure (Fig. S3). Moreover, STIM2 overexpression increased the expression of CD14 in THP1 cells treated with a small concentration of vitamin D (Fig. S4). In order to study the role of STIM2 in malignant myeloid cells, we used a knockdown (KD) strategy based on siRNA, directed against 2 different STIM2 sequences. siRNA was transduced using lipofection. Knockdown efficiency, assessed at protein level by western blot, was more than 60% (Fig. 2A). Using RT‐qPCR, we quantified the expression of the two STIM2 isoforms, STIM2.2 and STIM2.1, differing only by Exon 9. Although none of the targeted sequences was in that exon, we observed an increased STIM2.2/STIM2.1 ratio after siRNA transfection, showing that the efficacy on KD was predominant on STIM2.1, known to regulate negatively SOCE (Fig. 2B,C). In agreement with that, we performed Fura2 Ca^2+^ imaging assays in THP1 cells after siRNA transfection and observed on a large number of independent measurements, a trend toward increased SOC entry, peak, and plateau in comparison with the control, while the basal Ca^2+^ level and the response to Thapsigargin (that induces a Ca^2+^ release from ER storage) were similar (Fig. 2D–G). We then evaluated the effects of STIM2 KD on THP1 and OCI‐AML3 proliferation. Additionally, we used two shRNAi with a KD efficacy more than 90% at the protein level (Fig. 2H,I). In both cell lines, STIM2 KD drastically decreased the GFP^+^ cell number in comparison with cells transduced with the control vector (shSCRAMBLE) (Fig. 2J,K).

### 3.3 Transcriptomic deregulation in after STIM2KD in THP1 cells

**Fig. 3:**
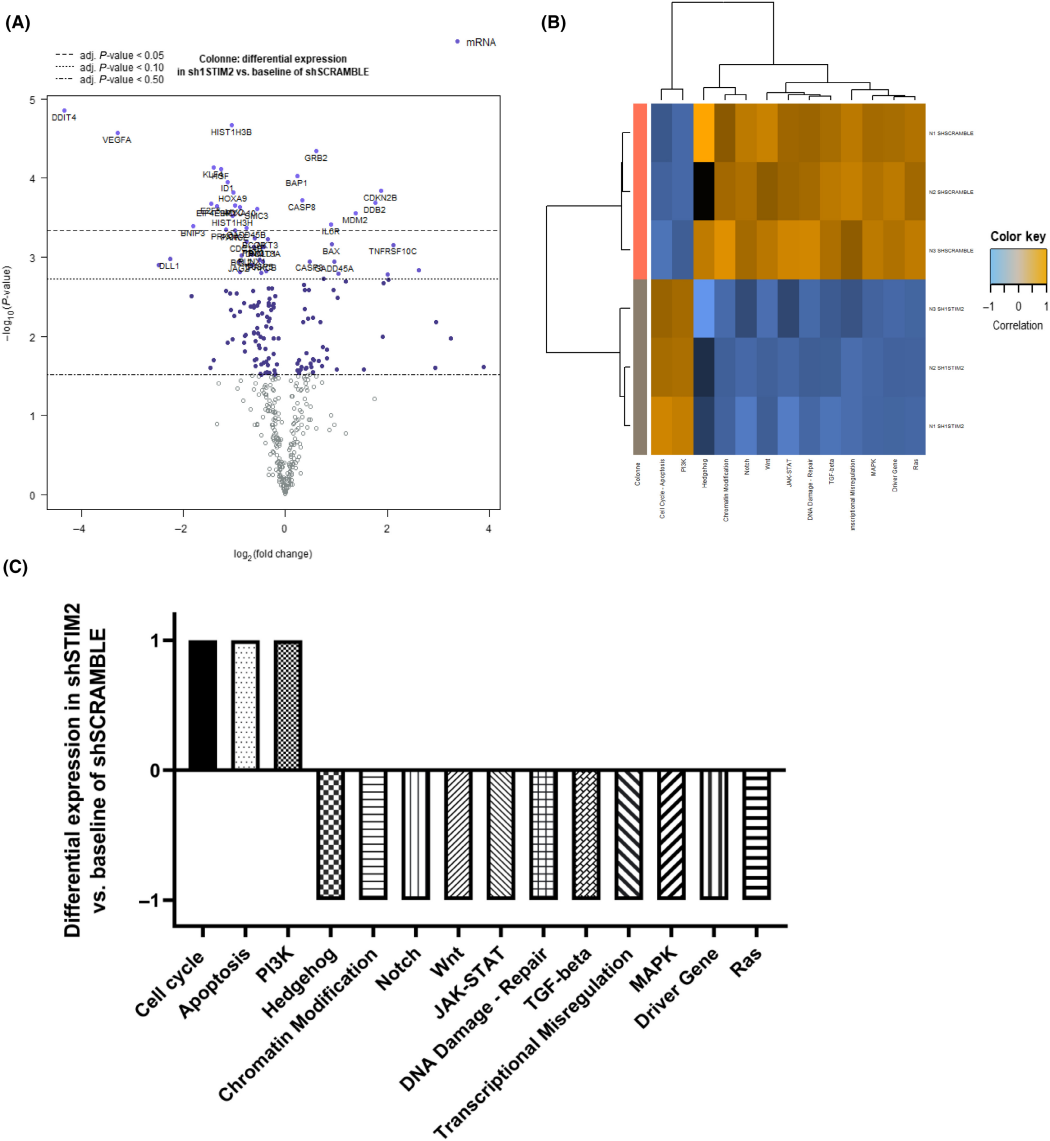
Transcriptomic deregulation after STIM2 knockdown in THP1 cells. Nanostring studies were carried out in the THP1 cell line. Knockdown (KD) STIM2 expression analysis on 770 genes using the PanCancer pathway panel of Nanostring technology. Volcanoplot shows the expression pattern of the entire gene set. *X*‐axis is log_2_(FC) and *Y*‐axis is −log_10_ (*P*‐value) for all covariates. The significant adjusted *P*‐value genes are at the top of the graph, above the first horizontal dashed line. (A) On the graph, only 26 genes significantly differentially expressed with an adjusted *P*‐value are shown. Heatmap showing the main pathways differentially expressed in cells after STIM2 KD versus control. The highly expressed pathways are in orange, whereas the lower ones are shown in blue. (B, C) The scores are viewed on the same scale using Z‐transformation (B) and in the representation with −1 for the lowest score and +1 for the highest score (C).

To decipher pathophysiological pathways involved in this decreased proliferation, we performed a transcriptomic study using the nCounter^®^ PanCancer Pathways Panel after STIM2 KD in THP1 cells. Out of the 395 genes from 770 target signals in this panel, 235 genes were differentially expressed with adj. *P*‐value < 0.1, including 197 genes with a significantly decreased expression (adj. *P*‐value < 0.05) in STIM2 KD condition. As shown in Fig. 3A,B, heatmap and volcano plots identified a distinctive gene expression signature after STIM2 KD. This signature was characterized by an upregulation of genes associated with apoptosis, cell cycle and chromatin modification and downregulation of expression of genes associated with DNA damage repair, and several crucial cell pathways such as hedgehog, JAK–STAT, MAPK, notch, PI3K, ras, TGF‐β, and wnt (see Fig. 3C and Table S7 for the list of differentially expressed genes).

### 3.4 STIM2 KD induced apoptosis in THP1 and OCI‐AML3 cells

**Fig. 4:**
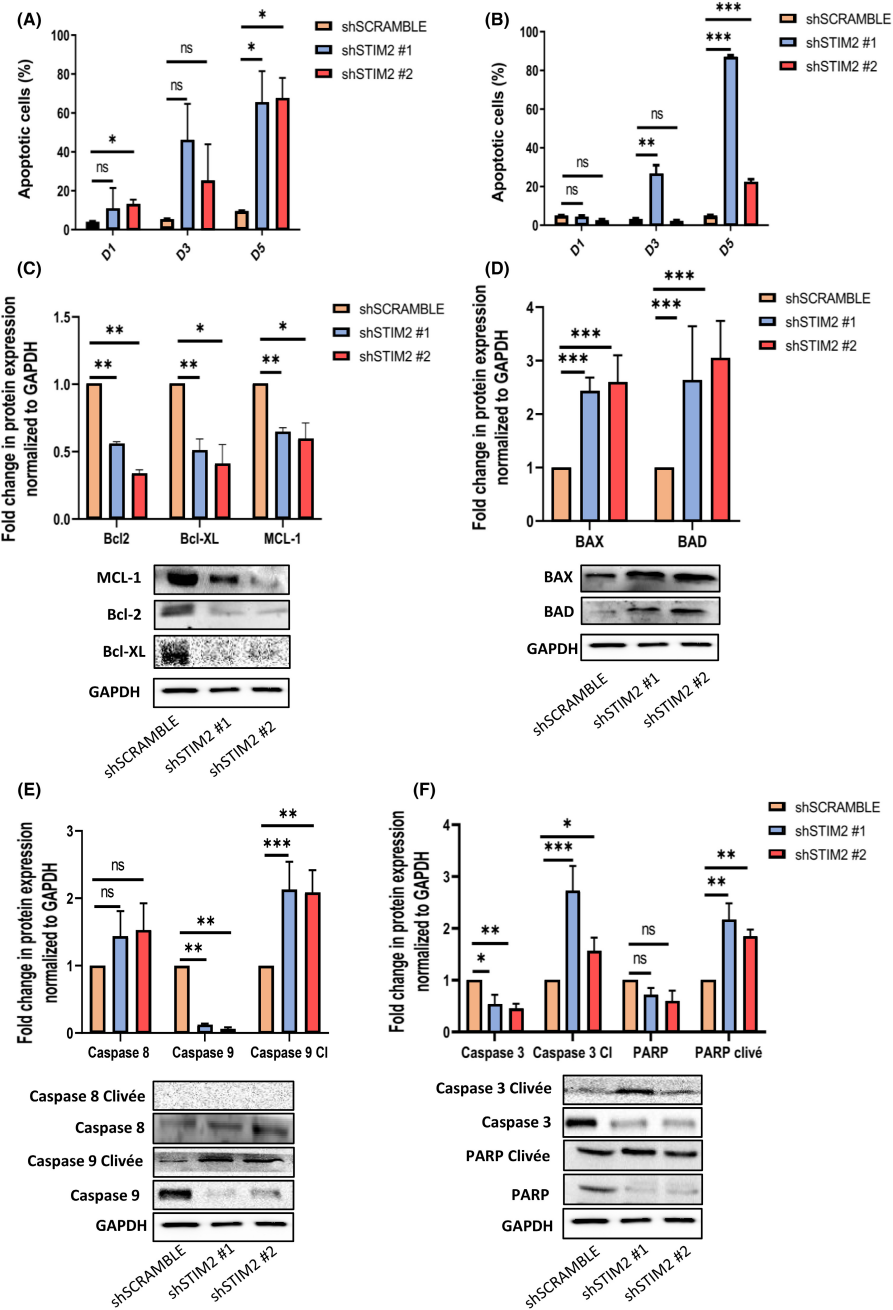
STIM2 knockdown induced apoptosis in THP1 and OCI‐AML3 cells. (A, B) Cell viability study was assessed in flow cytometry by annexin V/DAPI labeling and compared with shSCRAMBLE cells (Day 1—shSCRAMBLE: 4.14 ± 0.420; sh#1: 10.9 ± 10.5; sh#2: 13.3 ± 2.16) (Day 3—shSCRAMBLE: 5.43 ± 0.48; sh#1: 46.1 ± 18.6; sh#2: 25.5 ± 18.5) (Day 5—shSCRAMBLE: 9.68 ± 0.365; sh#1: 65.4 ± 16.1; sh#2: 67.6 ± 10.4) in THP1 (*n* = 3) (A) and in OCI‐AML3 (Day 1—shSCRAMBLE: 5.09 ± 0.175; sh#1: 4.49 ± 0.924; sh#2: 2.72 ± 0.655) (Day 3—shSCRAMBLE: 3.27 ± 0.667; sh#1: 26.7 ± 7.47; sh#2: 2.3 ± 0.524) (Day 5—shSCRAMBLE: 5.13 ± 0.242; sh#1: 87.2 ± 1.15; sh#2: 22.3 ± 2.52) (*n* = 3) (B). (C) In THP1 cell line, quantification of antiapoptotic proteins was performed by western blot (Bcl‐2—sh#1: 0.553 ± 0.0208; sh#2: 0.330 ± 0.0361) (Bcl‐XL—sh#1: 0.497 ± 0.0971; sh#2: 0.403 ± 0.150) (MCL‐1—sh#1: 0.642 ± 0.0351; sh#2: 0.590 ± 0.123) relative to GAPDH and compared with shSCRAMBLE (*n* = 3). (D) Quantification of proapoptotic proteins was performed by western blot (BAX—sh#1: 2.43 ± 0.252; sh#2: 2.6 ± 0.5) (BAD—sh#1: 2.63 ± 1.01; sh#2: 3.03 ± 0.718) relative to GAPDH and compared with shSCRAMBLE (*n* = 3). (E) Membrane and mitochondrial proteins were quantified by western blot (caspase‐8—sh#1: 1.42 ± 0.393; sh#2: 1.52 ± 0.407) (caspase‐9—sh#1: 0.103 ± 0.0351; sh#2: 0.0633 ± 0.0208) (cleaved (Cl) caspase‐9—sh#1: 2.11 ± 0.431; sh#2: 2.07 ± 0.347) relative to GAPDH and compared with shSCRAMBLE (*n* = 3). (F) Apoptosis effector proteins were quantified by western blot (caspase‐3—sh#1: 0.553 ± 0.185; sh#2: 0.457 ± 0.0874) (cleaved caspase‐3—sh#1: 2.71 ± 0.499; sh#2: 1.57 ± 0.248) (PARP—sh#1: 0.717 ± 0.136; sh#2: 0.593 ± 0.204) (cleaved PARP—sh#1: 2.16 ± 0.320; sh#2: 1.84 ± 0.140) relative to GAPDH and compared with shSCRAMBLE (*n* = 3). All numeric values are presented as the mean values ± standard error of the mean. *P*‐values are calculated using one‐way ANOVA, ***P < 0.001; **P < 0.01; *P < 0.05.

Considering the decreased cell proliferation together with the transcriptomic results obtained after STIM2 KD, we then assessed the consequences of this KD on cell viability and apoptosis in THP1 and OCI‐AML3 cells. In both cell lines, we observed a drastic increase at Day 5 (D5) of the Annexin V+/DAPI− and Annexin V/DAPI+ fractions in cells transduced with shSTIM2 lentiviruses (Fig. 4A,B). In THP1, STIM2 KD induced a drastic imbalance in the ratio between antiapoptotic and proapoptotic proteins with decreased expression of MCL‐1, BCL2, and Bcl‐XL (Fig. 4C) whereas the expression of BAX and BAD were significantly increased in comparison with shSCRAMBLE (Fig. 4D). This was associated with cleavage of caspase‐9, caspase‐3, and PARP, whereas caspase‐8 was not cleaved, arguing for STIM2‐induced activation of the mitochondrial intrinsic pathway of apoptosis (Fig. 4E,F). The same results were obtained in OCI‐AML3 cells (Fig. S5).

### 3.5 Blockage of cell cycle induced by STIM2 KD in THP1 and OCI‐AML3 cells

**Fig. 5:**
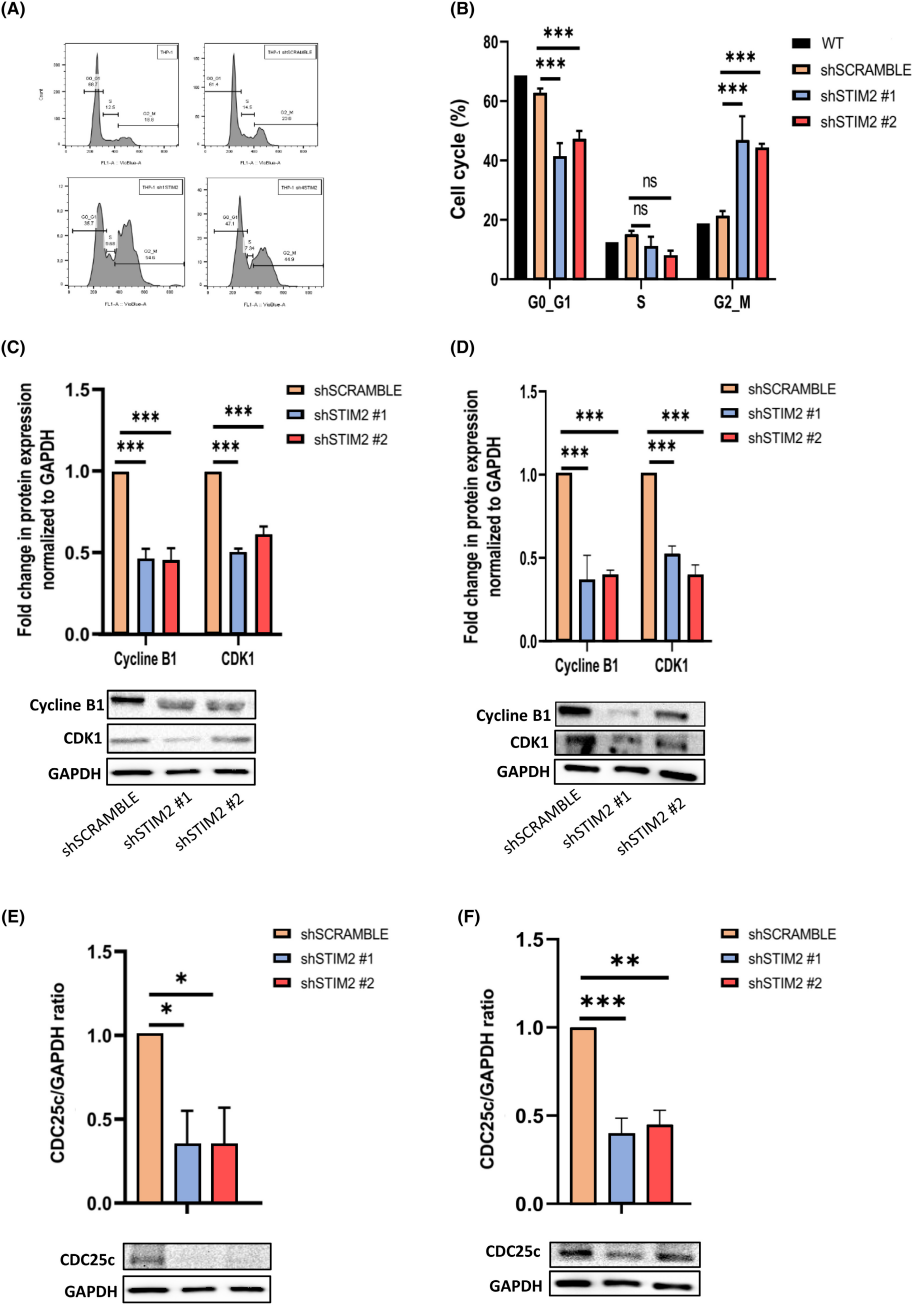
Blockage of cell cycle induced by STIM2 knockdown in THP1 and OCI‐AML3 cells. In THP1, the cell cycle was studied by flow cytometry (FCM) and western blot. (A, B) The FCM revealed an increase in the number of cells blocked in G2/M phase (shSCRAMBLE—19.77 ± 4.398; sh#1: 35.33 ± 6.047; sh#2: 32.80 ± 5.274) (*n* = 3). (C, D) In THP1, quantification of the key proteins of G2/M transition were performed by western blot (CDK1—sh#1: 0.507 ± 0.0231; sh#2: 0.617 ± 0.0493) (cyclin B1—sh#1: 0.467 ± 0.0611; sh#2: 0.460 ± 0.0721) relative to GAPDH (*n* = 3) (C) and in OCI‐AML3 (CDK1—sh#1: 0.527 ± 0.0451; sh#2: 0.393 ± 0.0643) (cyclin B1—sh#1: 0.370 ± 0.145; sh#2: 0.400 ± 0.0265) (*n* = 3) (D). (E, F) CDC25c was quantified by western blot in THP1 (CDC25c—sh#1: 0.350 ± 0.201; sh#2: 0.353 ± 0.216) (*n* = 3) (E) and in OCI‐AML3 (CDC25c—sh#1: 0.400 ± 0.0854; sh#2: 0.450 ± 0.0800) relative to GAPDH (*n* = 3) (F). All numeric values are presented as the mean values ± standard error of the mean. *P*‐values are calculated using one‐way ANOVA, ***P < 0.001; **P < 0.01; *P < 0.05.

Transcriptomic data on THP1 cells revealed that STIM2 KD strongly downregulated cell cycle‐associated genes (Fig. 3A,B). Assessing cell cycle progression by flow cytometry, we observed that STIM2 KD induced a significant accumulation of TPH1 cells in G2/M phase (Fig. 5A,B). By western blot, we observed a decrease in the expression of the Cyclin B1/CDK1 complex (Fig. 5C,D). This complex is regulated by the CDC25c phosphatase, which activates cell cycle regulatory kinases (CDKs) and thus controls the initiation of mitosis and proliferation [48]. Nanostring data identified the cell cycle as one of the most deregulated pathways in THP1 cells after STIM2 KD (Fig. 3B). Using western blot, we confirmed that CDC25c level decreased after STIM2 KD (Fig. 5E,F). Taken together, these data show that the decreased cell number observed in THP1 and OCI‐AML3 cells after STIM2 KD was related both to the activation of mitochondrial apoptosis and to an impairment of the G2/M transition associated with a low level of CDC25c and decreased expression of the Cyclin B1/CDK1 complex.

### 3.6 Apoptosis and cell cycle blockage are related to DNA double‐strand breaks and p53 induction

**Fig. 6:**
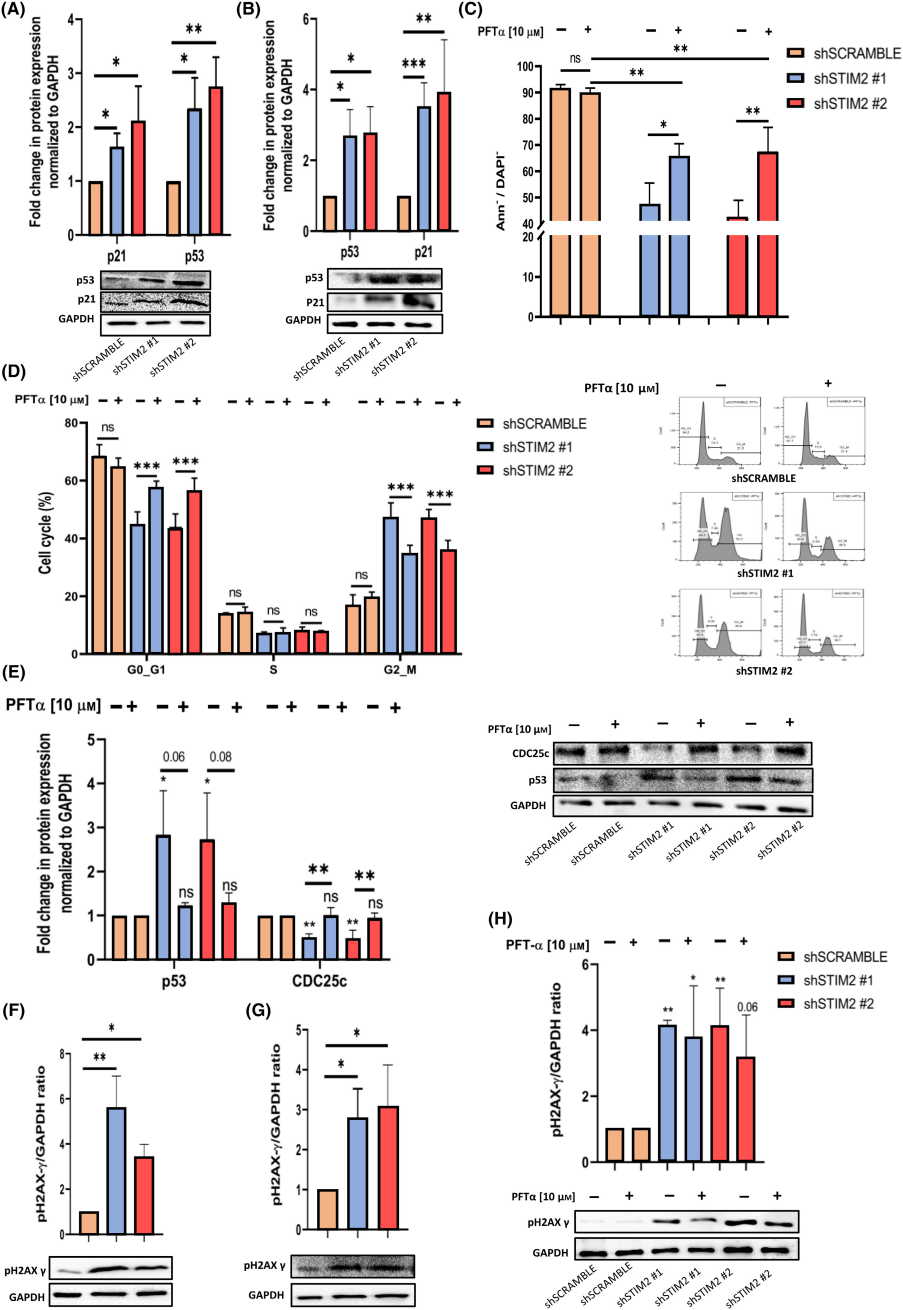
Apoptosis and cell blockage induced by STIM2 KD are related to DNA double‐strand breaks and p53 induction. (A, B) Expression of p53 and p21 in THP1 leukemic line with STIM2 KD was performed by western blot (p53—sh#1: 2.35 ± 0.560; sh#2: 2.75 ± 0.542) (p21—sh#1: 1.64 ± 0.214; sh#2: 2.12 ± 0.639) (*n* = 3) (A) and in OCIA‐AML3 (p53—sh#1: 2.69 ± 0.745; sh#2: 2.79 ± 0.735) (p21—sh#1: 3.52 ± 0.671; sh#2: 3.27 ± 0.439) relative to GAPDH (*n* = 3) (B), *P*‐values are calculated using one‐way ANOVA. (C) Cell viability was performed by Annexin/DAPI (Ann/DAPI) labeling in THP1 cells after STIM2 knockdown (KD), treated or not with pifithrin alpha (PFT‐α) (shSCRAMBLE −PFT‐α: 91.43 ± 1.582 – shSCRAMBLE +PFT‐α: 89.8 ± 1.9; sh#1 −PFT‐α: 47.27 ± 8.223 – sh#1 +PFT‐α: 65.67 ± 4.839; sh#2 −PFT‐α: 42.17 ± 6.772 – sh#2 +PFT‐α: 67.57 ± 9.097) (*n* = 3), *P*‐values are calculated using two‐way ANOVA. (D) Cell cycle analysis in THP1 cells after STIM2 KD, treated or not with PFT‐α was performed by flow cytometry (FCM) and compared with cells transduced with shSCRAMBLE (shSCRAMBLE −PFT‐α: 16.8 ± 3.64 – shSCRAMBLE +PFT‐α: 19.8 ± 1.69; sh#1 −PFT‐α: 47.2 ± 5.15 – sh#1 +PFT‐α: 34.7 ± 2.89; sh#2 −PFT‐α: 47.1 ± 2.95 – sh#2 +PFT‐α: 35.6 ± 3.69) (*n* = 5); *P*‐values are calculated using two‐way ANOVA. (E) Quantification of p53 and CDC25c at protein level was performed by western blot in THP1 cells after STIM2 KD treated or not with PFT‐α (p53—sh#1 −PFT‐α: 2.84 ± 0.9958 – sh#1 +PFT‐α: 1.237 ± 0.05508; sh#2 −PFT‐α: 2.737 ± 1.051 – sh#2 +PFT‐α: 1.297 ± 0.2196) (CDC25c—sh#1 −PFT‐α: 0.5033 ± 0.08083 – sh#1 +PFT‐α: 1.013 ± 0.1692; sh#2 ‐PFT‐α: 0.4833 ± 0.1850 – sh#2 +PFT‐α: 0.9533 ± 0.1069) (*n* = 3), *P*‐values are calculated using two‐way ANOVA. (F, G) p‐H2AXγ quantification was performed in THP1 by western blot (sh#1: 5.63 ± 1.38; sh#2: 3.45 ± 0.546) (*n* = 3) (F) and in OCI‐AML3 (sh#1: 2.8 ± 0.725; sh#2: 3.08 ± 1.04) relative to GAPDH (*n* = 3) (G), *P*‐values are calculated using one‐way ANOVA. (H) In THP‐1 cell line treated or not with PFT‐α, p‐H2AXγ quantification was performed by western blot (sh#1 −PFT‐α: 4.13 ± 0.176 – sh#1 +PFT‐α: 3.8 ± 1.55; sh#2 −PFT‐α: 4.14 ± 1.14 – sh#2 +PFT‐α: 3.6 ± 1.31) relative to GAPDH (*n* = 3), *P*‐values are calculated using two‐way ANOVA. All numeric values are presented as the mean values ± standard error of the mean. ***P < 0.001; **P < 0.01; *P < 0.05.

P53 is associated with apoptosis and cell cycle blockage by multiple pathways, including CDC25c regulation [49]. We then investigated whether the phenotype observed in THP1 cells after STIM2 KD was p53‐dependent. By western blot, we observed a strong induction of p53 at the protein level in shSTIM2‐transduced cells in comparison with controls together with increased expression of one of its transcriptional targets p21 (Fig. 6A,B). We exposed THP1 cells to Pifithrin‐α (PFT‐α), a transcriptional negative regulator of p53. PFT‐α, as expected, decreased p53 protein level and could partially revert the cell phenotype observed after STIM2 KD. Indeed, (a) it decreased the apoptotic rate (Fig. 6C), (b) it decreased the blockage in the G2/M phase of the cell cycle (Fig. 6D) and (c) it allowed the recovery of CDC25C expression (Fig. 6E). We assessed whether p53 induction was related to genotoxic stress. We quantified the double‐stranded DNA breakages (DSB) by western blotusing a p‐H2AXγ antibody. We observed a significant increase in the expression of p‐H2AXγ in THP1 and OCI‐AML3 cells after STIM2 KD (Fig. 6F,G). We then performed the same experiments in HL60, a myeloid cell line carrying a bi‐allelic deletion of p53, STIM2 KD‐induced DSB, but neither altered cell proliferation nor increased the apoptosis (Fig. S6). The same results were observed in K562 cells, in which p53 is also deleted (data not shown) and in THP‐1 treated with PFT‐α (Fig. 6H). Taken together, these data confirmed that the cell cycle blockage and apoptosis induced by a decreased level of STIM2 were related to p53 induction.

### 3.7 STIM2 involvement in normal monocytic differentiation

**Fig. 7:**
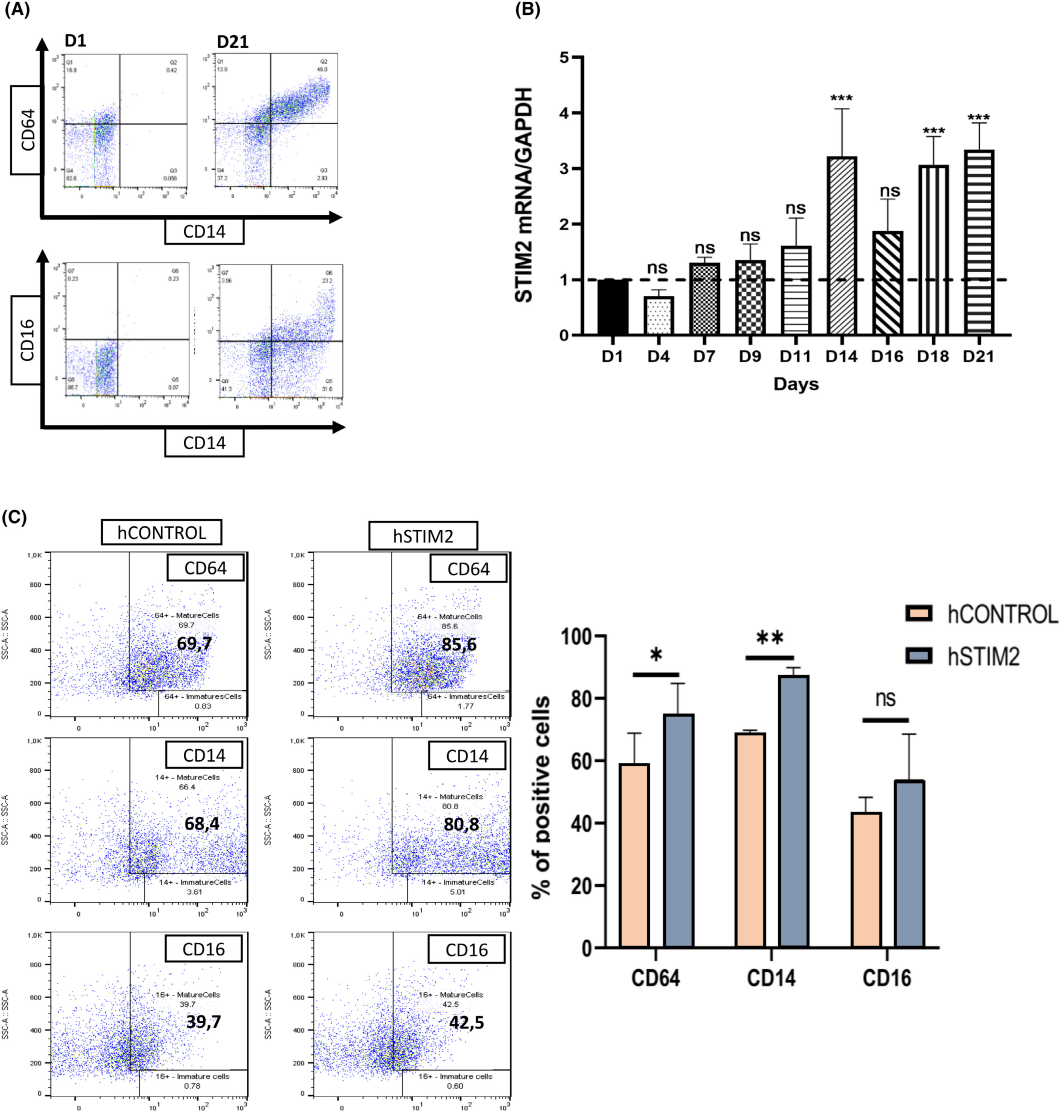
STIM2 expression during normal monocytic differentiation. *In vitro* monocyte primary cell differentiation from healthy donors. After magnetic sorting, CD34^+^ cells were cultured for 21 days in medium containing sequential cytokines. (A) Flow cytometry (FCM) dot plots assessing monocyte differentiation at early Day 1 (D1) and late Day 21 (D21) of CD64/CD14 and CD16/CD14. Dot plots are shown from 1 representative experiment (*n* = 3). (B) STIM2 mRNA expression determined by RT‐qPCR relative to HPRT expression, during monocyte differentiation. Statistical analysis was performed compared with D1. No significant change was seen on Days 4, 7, 9, 11, and 16 (*n* = 3), *P*‐values are calculated using one‐way ANOVA. (C) Study of monocyte differentiation in hematopoietic stem cells (HSCs) transfected with a lentiviral STIM2 cDNA expression vector. Differentiation was studied using FCM with specific markers (CD64–CD14–CD16) compared with control cells (hCONTROL) (CD64: hCONTROL: 58.4 ± 10.5; hSTIM2: 74.6 ± 10.2 – CD14: hCONTROL: 68.2 ± 1.6; hSTIM2: 87.4 ± 2.46 – CD64: hCONTROL: 43.2 ± 5.02; hSTIM2: 54 ± 14.5) (*n* = 3), *P*‐values are calculated using one‐way ANOVA. All numeric values are presented as the mean values ± standard error of the mean. ***P < 0.001; **P < 0.01; *P < 0.05.

**Fig. 8:**
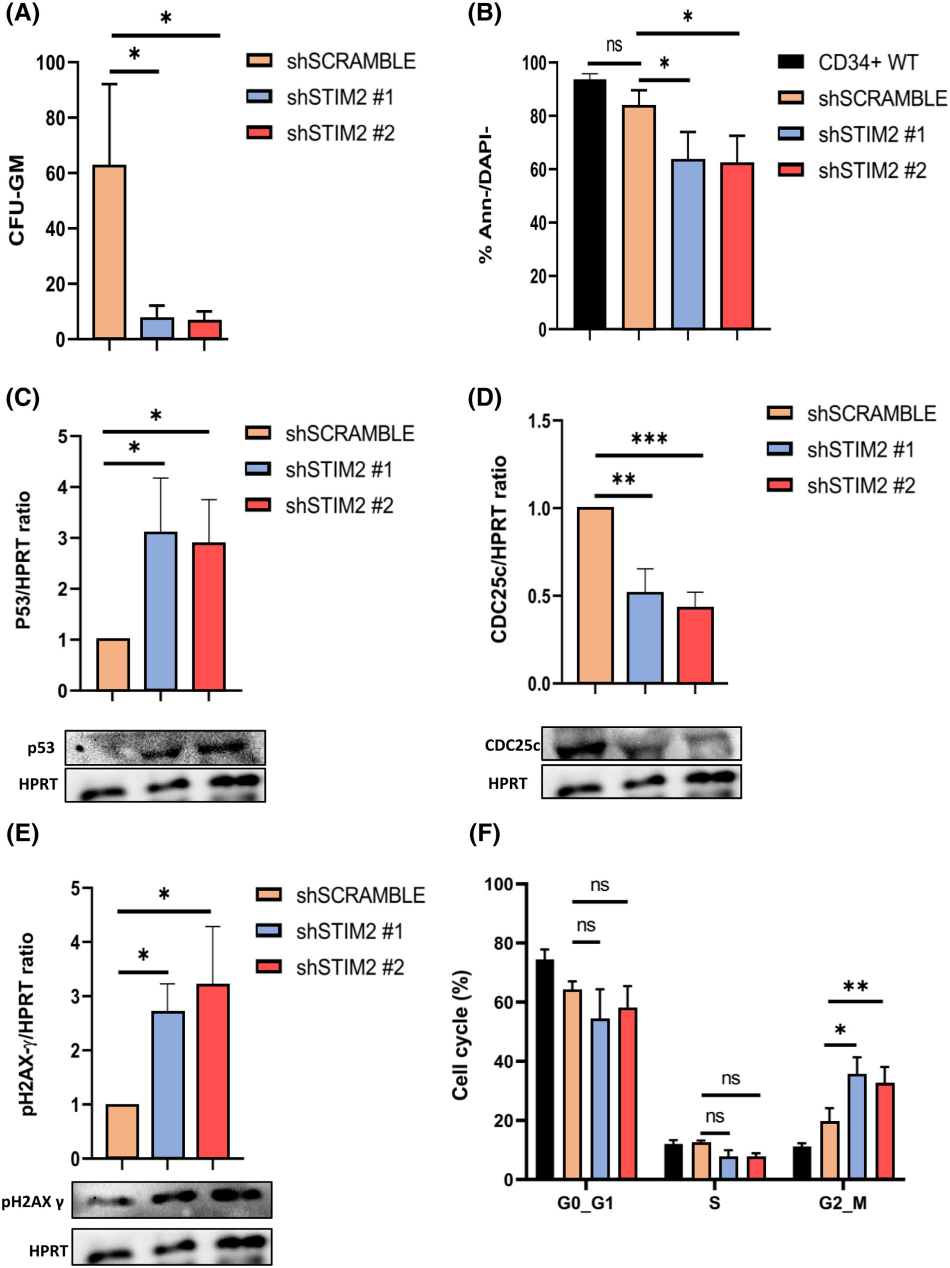
STIM2 knockdown during normal monocytic differentiation. STIM2 knockdown in primary cells induced monocyte differentiation. (A) Clonogenicity by methylcellulose was carried out over 14 days in 6‐well plates in triplicate. Colony‐forming unit granulo‐macrophagic (CFU‐GM) colonies were counted under an inverted microscope (shSCRAMBLE: 62.9 ± 29.3; sh#1: 7.87 ± 4.23; sh#2: 6.99 ± 3.06) (D) (*n* = 3), *P*‐values are calculated using one‐way ANOVA. (B) Cell viability was studied in FCM by annexin V/DAPI (Ann/DAPI) labeling (shSCRAMBLE: 83.6 ± 6.02; sh#1: 63.7 ± 10.2; sh#2: 62.5 ± 10.1) (*n* = 3), *P*‐values are calculated using two‐way ANOVA. (C) Quantification of p53 in primary monocytic cells after STIM2 knockdown (KD), assessed by western blot (sh#1: 3.13 ± 1.065; sh#2: 2.907 ± 0.8451) relative to HPRT (*n* = 3), *P*‐values are calculated using one‐way ANOVA. (D) Quantification of CDC25c, a key regulator of G2/M transition, in primary monocytic cells after STIM2 KD, as assessed by western blot (sh#1: 0.5167 ± 0.1380; sh#2: 0.4333 ± 0.08737) (*n* = 3), *P*‐values are calculated using one‐way ANOVA. (E) Quantification of p‐H2AXγ by western blot in primary monocytic cells after STIM KD (sh#1: 2.72 ± 0.506; sh#2: 3.21 ± 1.07) relative to HPRT (*n* = 3), *P*‐values are calculated using one‐way ANOVA. (F) Cell cycle analysis in primary monocytic cells after STIM2 KD assessed by FCM, showing an increase in cells at G2/M phase (shSCRAMBLE: 19.77 ± 4.398; sh#1: 35.33 ± 6.047; sh#2: 32.80 ± 5.274) (*n* = 3), *P*‐values are calculated using two‐way ANOVA. All numeric values are presented as the mean values ± standard error of the mean. ***P < 0.001; **P < 0.01; *P < 0.05.

To determine whether the role of STIM2 was restricted to malignant cells, we assessed whether these data obtained in leukemic cell lines were still relevant during normal myelopoiesis. We first quantified STIM2 expression at the RNA level during *in vitro* monocyte differentiation of sorted‐CD34^+^ cells obtained from apheresis from healthy donors (Fig. 7A). STIM2 mRNA increased from D14 to D21, in parallel with the appearance of monocytic markers at the cell surface (Fig. 7B). Moreover, transfection with a stable lentiviral STIM2 overexpression accelerated monocytic differentiation, as shown by an increased percentage of CD14^+^ cells (Fig. 7C). Lentiviral‐mediated STIM2 KD in CD34^+^ cells (Fig. S7) drastically reduced the CFU‐GM clonogenic potential in methylcellulose culture (Fig. 8A). Moreover, we observed the same phenotype induced by STIM2KD in the primary cells as in the malignant cell lines THP1 and OCI‐AML3. Indeed, we observed (a) decreased primary cell proliferation (Fig. 8B), (b) induction of p53 (Fig. 8C), (c) decreased cdc25c expression (Fig. 8D), increased p‐H2AXγ (Fig. 8E), and subsequent blockage in G2/M (Fig. 8F).

## 4 Discussion

Analysis of the TCGA and GTEx databases using the GEPIA2 tool revealed differential OS depending on the level of STIM2 expression. Indeed, patients who express the most STIM2 have reduced survival and a predominantly monoblastic and monocytic cytological subtype. This high STIM2 expression as a negative marker was also observed in glioblastoma [50], but this contrasts with data obtained in other tumor types, such as colorectal cancers where high STIM2 expression led to suppression of growth [51] and cholangiocarcinoma where low of STIM2 expression was associated with a poor prognosis [52]. These discrepant effects of STIM2 depending on the cancer type reflect its heterogeneous pattern of expression and function in different cell systems. In this study, using a shRNA‐mediated knockdown approach, we show that STIM2 is involved in genome integrity, cell cycle, and apoptosis control of primary cells and leukemic monocytic cell lines. Intracellular Ca^2+^ is known to play a key role in the regulation of proliferation and the cell cycle in both normal and malignant cells [53]. SOCEs have been widely described as being linked to increased proliferation [54]. Studies have mainly focused on STIM1, ORAI1, and TRPC1, showing that a reduction in SOCE influx was correlated with a parallel decrease in cell proliferation [55, 56, 57]. A few studies on STIM2 have been carried out, with conflicting results on SOCE measurement and cell responses. In HUVECs and pulmonary artery smooth muscle cells, a decreased STIM2 expression led to a loss of proliferative capacity [58, 59]. We observed here a similar effect in both primary hematopoietic cells and leukemic cells. First, STIM2 KD induced apoptosis by the mitochondrial intrinsic pathway, with an increased ratio between proapoptotic and antiapoptotic proteins. Second, it let to dysregulation of the cell cycle, characterized by G2/M‐phase arrest, with a decrease in cell cycle regulators controlling the G2/M transition. Indeed, in order to enter into mitosis from G2, cells must activate CDK1, which binds to cyclin B [60]. This activation depends on the phosphatase CDC25c, which removes a phosphate group from CDK1 [61]. The expression of CDK1, cyclin B1, and CDC25c were all drastically decreased following STIM2 KD.

We show here that both cell cycle blockage and the apoptotic response to STIM2 KD occurred through genomic stress (assessed by quantification of DSB) and p53 activation. The link between DNA breakage, H2AXγ phosphorylation, and blockage in G2/M has been reported in other cell types, after low‐dose irradiation or exposure to toxins such as Benzo (a) pyrene [62]. p‐H2AXγ allows recruitment at DNA double‐strand breakpoints of proteins involved in DNA repair, activates directly the p53 pathway [63], which induces p21 [63], inhibits the CDK1‐cyclin B1 complex [48, 49], and represses CDC25c phosphatase [49], leading to cell blockage in G2/M to prevent defective mitosis. Of note, p53 is mutated in THP1 cells but is still expressed and functional, in agreement with other reports [64, 65, 66]. The central role of p53 in response to DNA stress mediated by STIM2 KD is highlighted by (a) the reversal of apoptosis and cell cycle blockage after cell exposure to the p53 inhibitor PFT‐α and (b) the absence of a phenotype in two cell lines defective for p53, HL60, and K562. Of note, it could be claimed that p‐H2AXγ is a consequence and not a cause of p53 stabilization and of the subsequent activation of the apoptotic cascade since DNA ladder formation during apoptosis requires JNK‐dependent phosphorylation of H2AXγ in cooperation with the caspase‐3/CAD pathway [67]. However, in leukemic and normal hematopoietic cells, PFT‐α reversed cell cycle blockage and apoptosis but not p‐H2AXγ induction, showing that genomic stress occurred upstream of p53 induction in STIM2 KD conditions. The cell response to p53 induction is either a reversible cell cycle blockage or apoptosis, depending on the balance between pro‐ and antiapoptotic proteins that determines a ‘threshold’ beyond which cells will die [68, 69, 70]. In OCI‐AML3 and THP1 cells after STIM2 KD, this threshold is low as a consequence of decreased expression of MCL‐1, a p53 target, as well as BCL2 and Bcl‐XL, and increased expression of BAX and BAD. One can assume that this low ‘trigger’ drives the massive apoptosis that we observed in response to the cell cycle blockage induced by p53 activation. Notably, the simultaneous decrease in BCL2 and MCL‐1 levels induced by STIM2 KD is particularly interesting and relevant in therapeutics considering that high expression of MCL‐1 is involved in the resistance of AML cells to targeted therapy, such as BCL2 Inhibitors [71].

To build a functional link between STIM2, Ca^2+^ response, and DNA stress, we measured Ca^2+^ entry through SOCE after Thapsigargin exposure. One could assume that STIM2 KD would decrease the intracellular Ca^2+^ level in response to ER depletion. In contrast to another report [72], we observed increased Ca^2+^ entry in response to Thapsigargin after STIM2 KD. We first ruled out any compensatory mechanism by increased STIM1 expression since its level was stable after STIM2 KD (not shown). However, since STIM1 and STIM2 interact and since STIM1 activates ORAI more efficiently, we cannot rule out that STIM2 KD promotes the formation of STIM1 homomeric complexes capable of recruiting and stimulating SOC entry more efficiently [73]. Moreover, the effect of STIM2 modulation on SOCE is conflicting in the literature [13, 74, 75]. This may be due to the coexistence of different STIM2 isoforms, STIM2.1 and STIM2.2, which differ in their expression pattern and function on SOCE. Indeed, the largely expressed STIM2.2 isoform enhances SOCE whereas STIM2.1, which contains a new sequence within the CAD domain [76, 77], represses SOCE through abrogation of its interaction with ORAI. Despite the fact that all the shRNAs that we used targeted sequences outside the 8 residues specific to STIM2.1, the 2.2/2.1 STIM2 ratio was increased after shSTIM2 transduction, explaining the positive effect on SOCE. BAPTA, an intracellular Ca^2+^ chelator, was able to decrease STIM2 KD‐mediated apoptosis in THP1 cells (Fig. S8), arguing for a link between STIM2 KD, deregulated Ca^2+^ signaling and cell death. Furthermore, the AUC results for Thapsigargin response showed that transfections do not induce a reticular calcium stress and therefore cannot explain any apoptotic effects. Of note, the level of SOCE observed in the siCONTROL condition was low. This was not due, in contrast with other cell types harboring a weak SOC entry, to a low expression of ORAI channels. Our hypothesis is that, under basal conditions, SOCE activity in THP1 is tightly regulated, allowing cells to proliferate and survive. Removing this control by increasing the STIM2.2/STIM2.1 ratio increases drastically the cytosolic Ca^2+^ level, mediates the DNA stress, and subsequently apoptosis and cell cycle blockage. However, the exact nature of the functional link between the altered SOCE response in monocytic cells and DNA stress is lacking.

In addition to these effects on the cell cycle and apoptosis, we pointed out a potential role of STIM2 in monocytic differentiation, as already described in naïve CD8^+^ T‐cell maturation into cytotoxic terminal effector cells [78, 79]. STIM proteins are expressed in monocyte/macrophage function, as shown by KO mouse models. Sogkas et al. found that STIM1 was involved in phagocytosis, whereas STIM2 was involved in cell migration and apoptosis, particularly in the production of pro‐inflammatory cytokines [80], although these results were not confirmed by other teams [81]. In our study, we observed trends toward monocytic differentiation in AML expressing higher STIM2 levels and increased STIM2 expression during *in vitro* monocytic differentiation of CD34^+^ and leukemic cell lines. Moreover, STIM2 KD impaired CD14 expression whereas STIM2 overexpression increased its expression in THP1 cells exposed to low level of vitamin D. Once again, the functional link between SOCE deregulation after STIM2 KD and monocytic differentiation is lacking. However, such mechanism remains plausible considering the data in the literature [82, 83]. For example, differentiation of the myelomonocytic cell line U937 toward macrophage by dibutyryl‐cAMP was associated with upregulation of Calcium release‐activated calcium channel (CRAC) activity. Thapsigargin‐induced Ca^2+^ release from ER calcium was higher in differentiated U937 than their undifferentiated counterpart [82]. Macrophages and monocytes express ORAI 1, 2, and 3 as shown in transcriptomic studies, and CRAC is involved during macrophage activation and ROS production. ORAI1 may be the most abundant, while ORAI3 may induce negative feedback in order to prevent cells from oxidative damages. In THP1 cells, exposure to oxidized LDL increased ORAI‐dependent Ca^2+^ intake, whereas Ca^2+^ chelation or ORAI1 inhibition decreased cell formation. Chemical inhibition of ORAI1 in apoE−/− mice drastically decreased atherosclerosis formation induced by a high‐cholesterol diet [84]. Taken together, these data, including ours, suggest a particular role of SOCE and Ca^2+^ signaling in monocytic/macrophage differentiation and function.

## 5 Conclusion

In conclusion, we describe here STIM2, the ‘forgotten’ member of SOCE, as a new actor in the proliferation, survival, and differentiation of human normal and malignant monocytic cells. Considering (a) the association of high STIM2 expression with an adverse prognosis in AML and (b) the association of STIM2 with AML harboring monocytic/myelomonocytic differentiation, STIM2 may represent an interesting protein to target in these types of leukemia in the future.

## Conflict of interest

The authors declare no conflict of interest.

## Author contributions

SD, LC, GJ, FH, JH, JL, DL, and HO‐H performed the experiments. RI performed figure and statistical analysis for ALFA0702 cohort. J‐PM, EP, FH, TB, and LG designed the experiments. SD, TB, and LG wrote the manuscript.

### Peer review

The peer review history for this article is available at https://www.webofscience.com/api/gateway/wos/peer‐review/10.1002/1878‐0261.13584.

## Supporting information


**Fig. S1.** STIM2 expression in ALFA0702 cohort.
**Fig. S2.** Vitamin D induces differentiation on THP‐1 and OCI‐AML3 cell line.
**Fig. S3.** STIM2 expression in the monocytic lineage.
**Fig. S4.** STIM2 overexpression induces differentiation in lineage and normal cell line.
**Fig. S5.** Mitochondrial apoptosis in OCI‐AML3 cell line.
**Fig. S6.** Effect of STIM2 knockdown in HL60 cell line.
**Fig. S7.** STIM2 knockdown in normal hematopoietic stem cell.
**Fig. S8.** BAPTA decreases STIM2 KD‐mediated apoptosis in THP‐1 cells.
**Table S1.** Characteristics and references of antibodies used for flow cytometry.
**Table S2.** Location and targeted sequences of the 2 shRNA anti‐STIM2.
**Table S3.** hRNA design vector.
**Table S4.** Location and targeted sequences of the 2 siRNA anti‐STIM2.
**Table S5.** List of primers used for RT‐qPCR.
**Table S6.** Characteristics and reference antibodies used for western blot.

**Table S7.** Transcriptomic deregulation genes.

## Data Availability

The TCGA dataset used and analyzed during the current study was available from the corresponding author on reasonable request. Nanostring datasets generated and analyzed during the current study are included in the published article (Table S7).
