# Supplementary material for: STIM2 is involved in the regulation of apoptosis and the cell cycle in normal and malignant monocytic cells
Source: Mol Oncol. 2024 Jan 17;18(6):1571–92. doi: 10.1002/1878-0261.13584 (PMC11161727; doi:10.1002/1878-0261.13584)
Supplement: Supplementary file 1 — Fig. S1. STIM2 expression in ALFA0702 cohort. Fig. S2. Vitamin D induces differentiation on THP‐1 and OCI‐AML3 cell line. Fig. S3. STIM2 expression in the monocytic lineage. Fig. S4. STIM2 overexpression induces differentiation in lineage and normal cell line. Fig. S5. Mitochondrial apoptosis in OCI‐AML3 cell line. Fig. S6. Effect of STIM2 knockdown in HL60 cell line. Fig. S7. STIM2 knockdown in normal hematopoietic stem cell. Fig. S8. BAPTA decreases STIM2 KD‐mediated apoptosis in THP‐1 cells. Table S1. Characteristics and references of antibodies used for flow cytometry. Table S2. Location and targeted sequences of the 2 shRNA anti‐STIM2. Table S3. hRNA design vector. Table S4. Location and targeted sequences of the 2 siRNA anti‐STIM2. Table S5. List of primers used for RT‐qPCR. Table S6. Characteristics and reference antibodies used for western blot. [file MOL2-18-1571-s001.pdf]

Supplementary Table 1: Characteristics and references of antibodies used for flow cytometry

Target molecule	Host species	Clone	Dilution	Conjugated	Source
**CD14**	Human	REA55	1:100	PE-Vio770	Miltenyi Biotec
**CD64**	Human	22	1:40	FITC	Beckman Coulter
**CD11b**	Human	Bear1	1:40	PE	Beckman Coulter
**CD34**	Mouse	581	1:40	APC	BD Pharmingen ™
**CD16**	Human	REA423	1:100	PE	Miltenyi Biotec
**CD64**	Human	REA978	1:100	APC-Vio770	Miltenyi Biotec

Supplementary Table 2: Location and targeted sequences of the 2 shRNA anti STIM2

sh-STIM2 clone	Clone ID	Target sequence	Position
**#1**	TRCN0000154373	CCCTGCGCTTTATCGAAATGA	Exon #11
**#2**	TRCN0000150632	GCACGAACCTTCATTTATGAT	Exon #5

Supplementary Table 3: hRNA design vector

Clone	Clone ID	Vector Size	Cloning Host
**hCONTROL**	pLV[Exp]-EGFP:T2A:Puro-EF1A>ORF_Stuffer	9623 bp	Ampicillin
**hSTIM2**	pLV[Exp]-EGFP:T2A:Puro-EF1A>hSTIM2	11639 bp	Ampicillin

Supplementary Table 4: Location and targeted sequences of the 2 siRNA anti STIM2

si-STIM2 clone	Target sequence
#1	CACUGAAGUAGAAGUGCAAUACUA
#2	CCAGAAUAAGCAGCAUCCCACAUGA

Supplementary Table 5: List of primers used for RT-qPCR

Gene	Orientation	Sequence
**GAPDH**	Forward<br>Reverse	- AAGGTGAAGGTCGGAGTCAA -<br>- CTTGACGGTGCCATGGAATT -
**STIM2**	Forward<br>Reverse	- TGACAGATCCCTGCATGTCAC -<br>- TCTGTGCAGATGGCTGTGTTT -
**STIM2**<br>**STIM2.1**<br>**STIM2.2**	Forward<br>Reverse<br>Reverse	- CTCTGAAAAAGGCCGAAAA -<br>- TGAAGCAGCAACCTCATCTTT -<br>- TTTTCTGCCTCATCTTTAGCAA -
**CD14**	Forward<br>Reverse	- AAGACTTATCGACCATGGAGCG -<br>- CTTCATCGTCCAGCTCACAAG -
**CD11b**	Forward<br>Reverse	- AGAGAACGCAAGGGGCTTC -<br>- GTAGTCGCACTGGTAGAGGC -
**CD64**	Forward<br>Reverse	- CGACCCCCAGCTACAGAATC -<br>- CTGGCATCCACATCCCTCTCATC -
**CCR-2**	Forward<br>Reverse	- TTGGTTCAGTTGCTGAGAAGC -<br>- GGATGTACTGGGGAAATGCG -
**CXCR-1**	Forward<br>Reverse	- CAGCTCCTACTGTTGGACACA -<br>- ATCCCACATCTGTGGATCTGT -
**LYSOZYME**	Forward<br>Reverse	- AGGTGTGAGTTGGCCAGAAC -<br>- ATCAGTGCTTCTGTCTCCAGC -

Supplementary Table 6: Characteristics and reference antibodies used for WesternBlot

Antibodies Targets	Antibodies species	References
**Anti-human STIM2**	Rabbit	Abcam (59342)
**Anti-human Caspase8**	Rabbit	Cell signaling (9496S)
**Anti-human Caspase9**	Rabbit	Cell signaling (9502)
**Anti-human Caspase9 Clivée**	Rabbit	Cell signaling (7237S)
**Anti-human Caspase3**	Rabbit	Cell signaling (9664S)
**Anti-human PARP**	Rabbit	Cell signaling (9542S)
**Anti-human BAX**	Rabbit	Cell signaling (2772)
**Anti-human BAD**	Rabbit	ProteinTech (10435-1-AP)
**Anti-human BCL-xL**	Rabbit	Cell signaling (2762)
**Anti-human Bcl2**	Rabbit	Invitrogen (100/D5)
**Anti-human MCL-1**	Rabbit	Cell signaling (4572S)
**Anti-human CDC25c**	Rabbit	Cell signaling (4688S)
**Anti-human CDK1**	Rabbit	Abclonal (A0220)
**Anti-human Cycline B1**	Rabbit	Cell signaling (D5C10)
**Anti-human p53**	Rabbit	Cell signaling (2527S)
**Anti-human p21**	Rabbit	Santacruz (397)
**Anti-human p-Histone H2A.X**	Mouse	Millipore (JBW301)
**Anti-human HPRT**	Rabbit	Proteintech (15059-1-AP)
**Anti-human GPADH**	Mouse	Santacruz (32233)

Supplementary Table 7: Transcriptomic deregulation genes

Fichier CSV
Microsoft Excel

Supplementary Figure 1

A

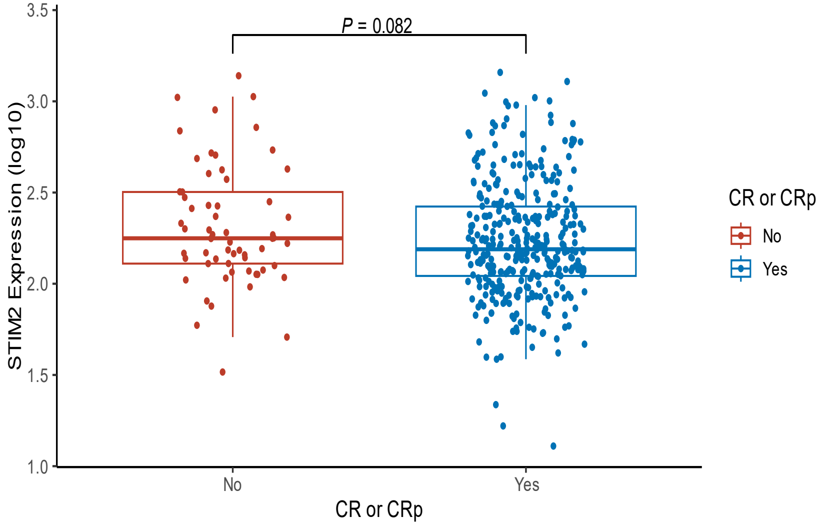

B

Variable		N	Odds ratio		p
**STIM2_high**		407		0.50 (0.25, 0.97)	0.04
**eln17_f**	Favorable	152		Reference	
	Intermediate	130		0.16 (0.06, 0.41)	<0.001
	Adverse	125		0.06 (0.02, 0.15)	<0.001
**log10(gbi)**		407		0.67 (0.41, 1.11)	0.12
**clara**		407		30.20 (8.85, 189.97)	<0.001

Supplementary Figure 1: STIM2 expression in ALFA0702 cohort. (**A**) Univariable analysis of STIM2 expression in patients achieving CR/CRp or not. (**B**) Lower odds of reaching complete remission of higher STIM2 expression expressed as a continuous variable.

Supplementary Figure 2

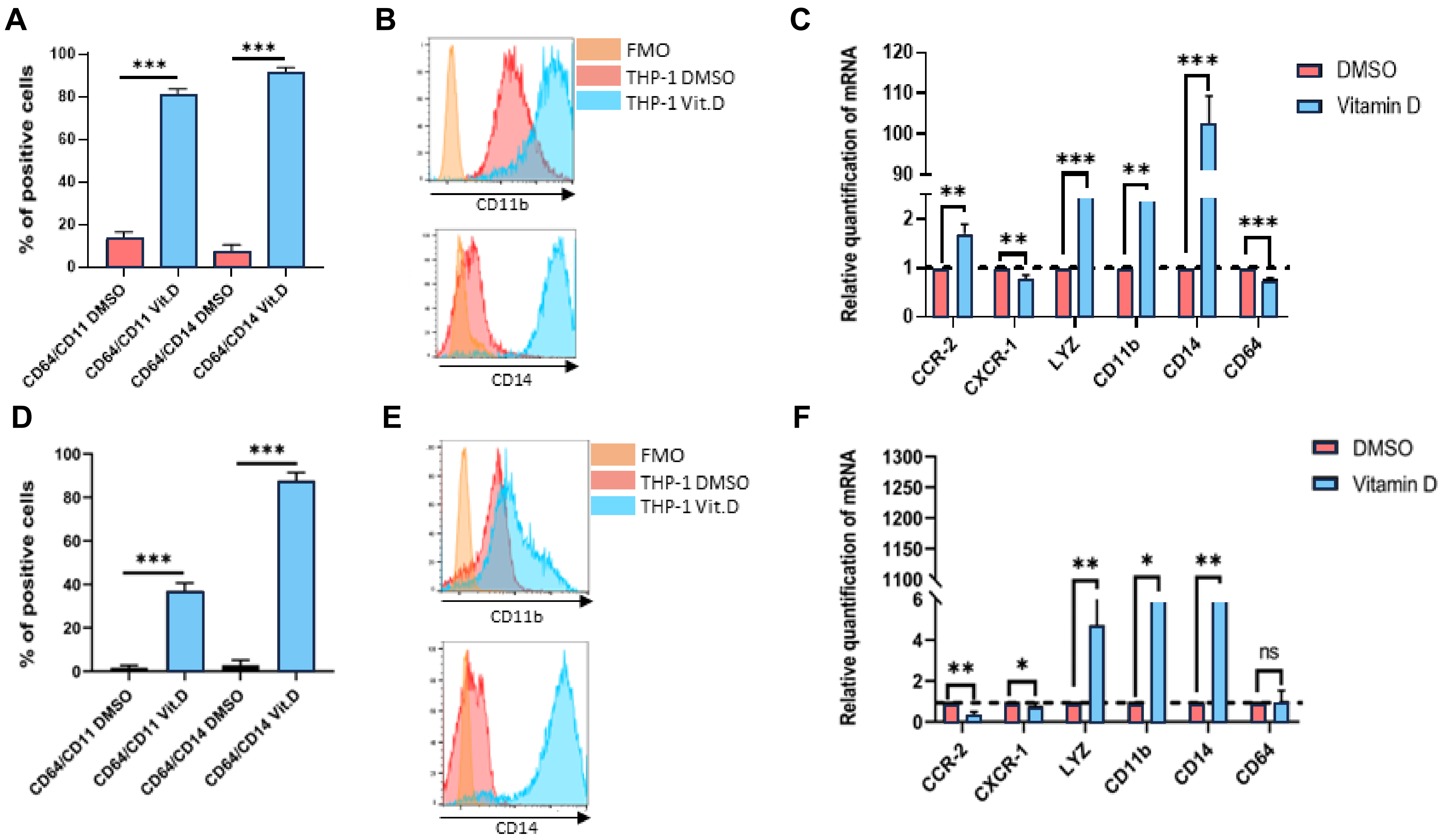

Supplementary Figure 2: Vitamin D induce differentiation on THP-1 and OCI-AML3 cell line. (**A**) FCM quantification of CD14, CD11b and CD64 expression in THP-1 treated with 1μM vitamin D as described in materials and methods (n=6). (**A**-**B**) FCM and representative multiparametric flow cytometry histograms showing increased expression of CD14 (91,8 ± 1,98%) and CD11b (80,8 ± 2,88%) after treatment with vitamin D as compared to DMSO. (**C**) Monocyte differentiation markers genes CCR-2 (1.70±0.115), CXCR-1 (0.78±0.043), LYZ (3.77±0.217), CD11b (13.4 ± 1,59), CD14 (103 ± 3,84), CD64 (0,77 ± 0,0120) (determined by RT-qPCR) relative to GAPDH expression (n=3). (**D**) FCM quantification of CD14, CD11b and CD64 expression in OCI-AML3 treated with 1μM vitamin D (n=3). (**D**-**E**) FCM and representative multiparametric flow cytometry histograms showing increased expression of CD14 (87,73 ± 3,73%) and CD11b (37 ± 3,623%) after treatment with vitamin D as compared to DMSO. (**F**) Monocyte differentiation markers genes CCR-2 (0,360 ± 0,154), CXCR-1 (0,80±0,139), LYZ (4,74±1,35), CD11b (20,7 ± 11.9), CD14 (968 ± 74,2), CD64 (0,937 ± 0,590) was performed by RT-qPCR relative to GAPDH expression (n=3). ****P<0,001; **P<0,01; *P<0,05*

Supplementary Figure 3

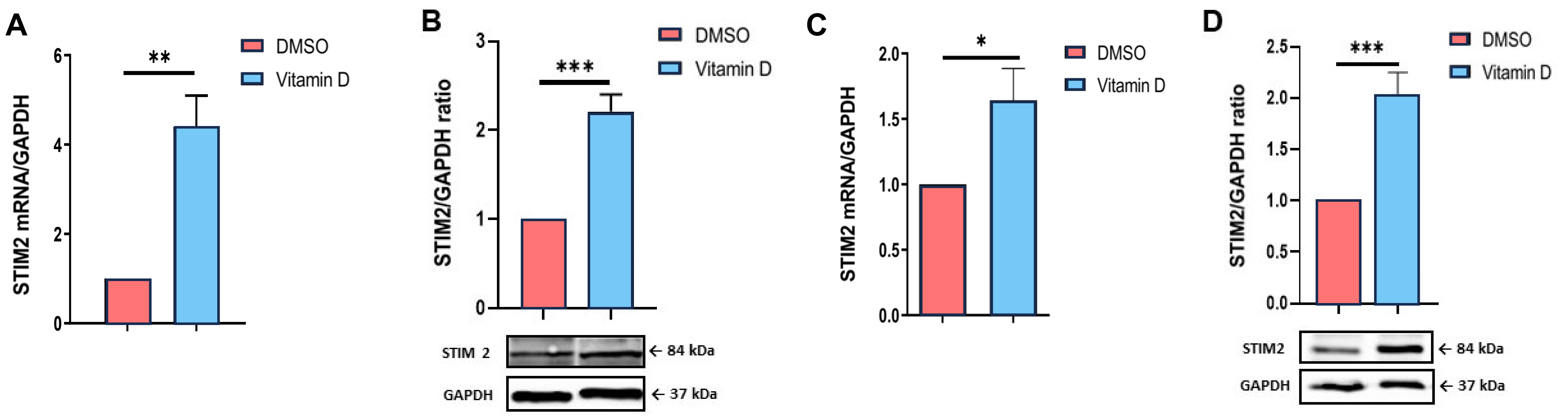

Supplementary Figure 3: STIM2 expression in the monocytic lineage. RT-qPCR and Western Blot were performed after 72h of culture in medium containing vitamin D compared to dimethylsulfoxide (DMSO). (**A**) In THP-1, STIM2 mRNA expression (4,40 ± 0,7) (determined by RT-qPCR) relative to GAPDH expression during monocyte differentiation in THP-1 cell line (n=3). (**B**) Quantification of STIM2 at protein level was performed by Western Blot (2.21±0.11) during monocyte differentiation (n=3). (**C**) In OCI-AML3, STIM2 mRNA expression (1,643 ± 0,2417) was performed by RT-qPCR relative to GAPDH expression during monocyte differentiation (n=3). (**D**) Quantification of STIM2 at protein level was performed by Western Blot (2,040 ± 0,13) during monocyte differentiation (n=3). ****P<0,001; **P<0,01; *P<0,05*

Supplementary Figure 4

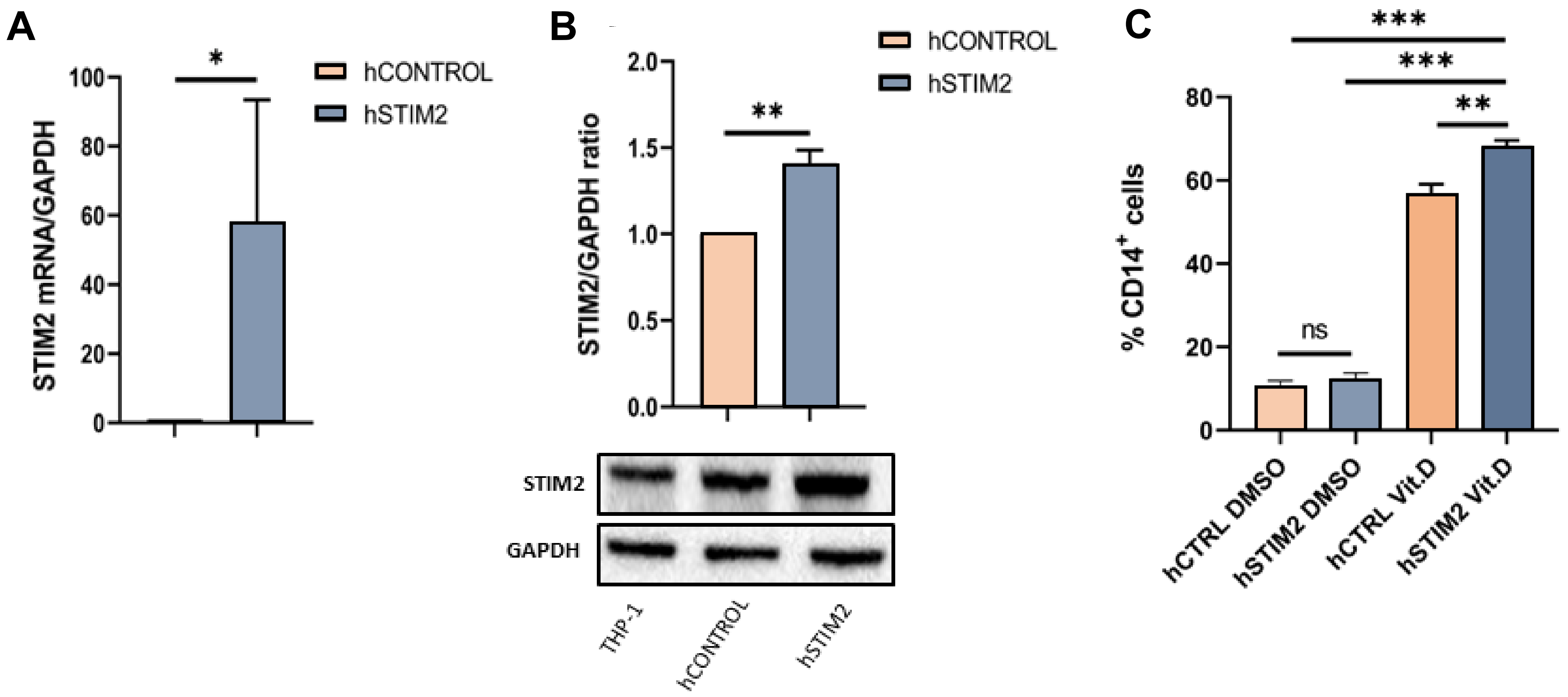

Supplementary Figure 4: STIM2 overexpression induce differentiation in lineage and normal cell line. (**A**-**B**) STIM2 quantification in THP-1 line was performed by RT-qPCR (58,1 ± 35,3) (**A**) (n=4) and Western Blot (**B**) (n=4) compared with control cells (hCONTROL). (**C**) Monocytic differentiation was compared by FCM between cells overexpressing STIM2 and cells transfected with the empty vector in the presence of vitamin D at 1 nM or DMSO (hCONTROL DMSO: 10.4 ± 1.57; hCONTROL Vitamin D: 56.6 ± 2.45 - hSTIM2 DMSO: 12.3 ± 1.5; hSTIM2 Vitamin D: 68.2 ± 1.46) (n=3). ****P<0,001; **P<0,01; *P<0,05*

Supplementary Figure 5

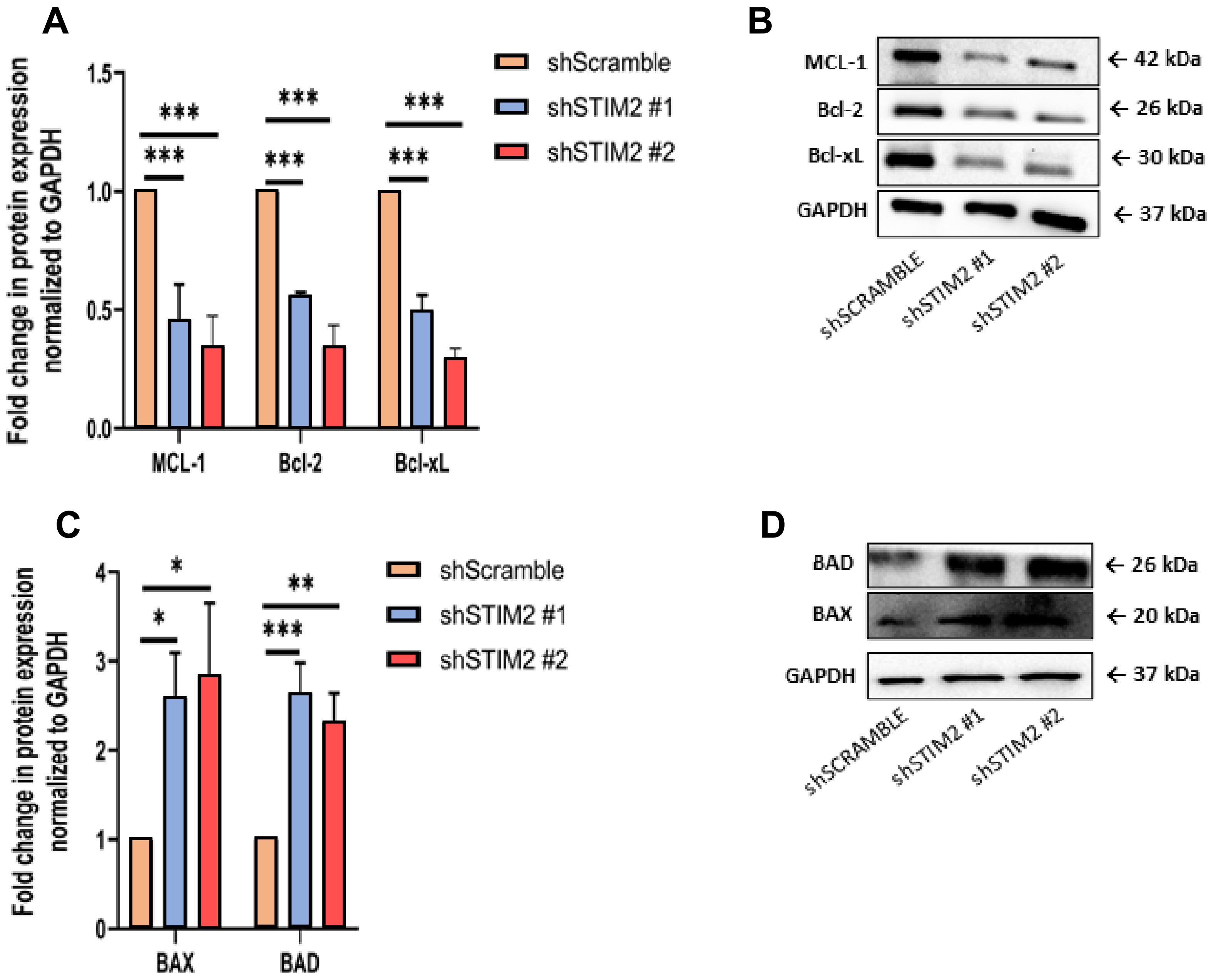

Supplementary Figure 5

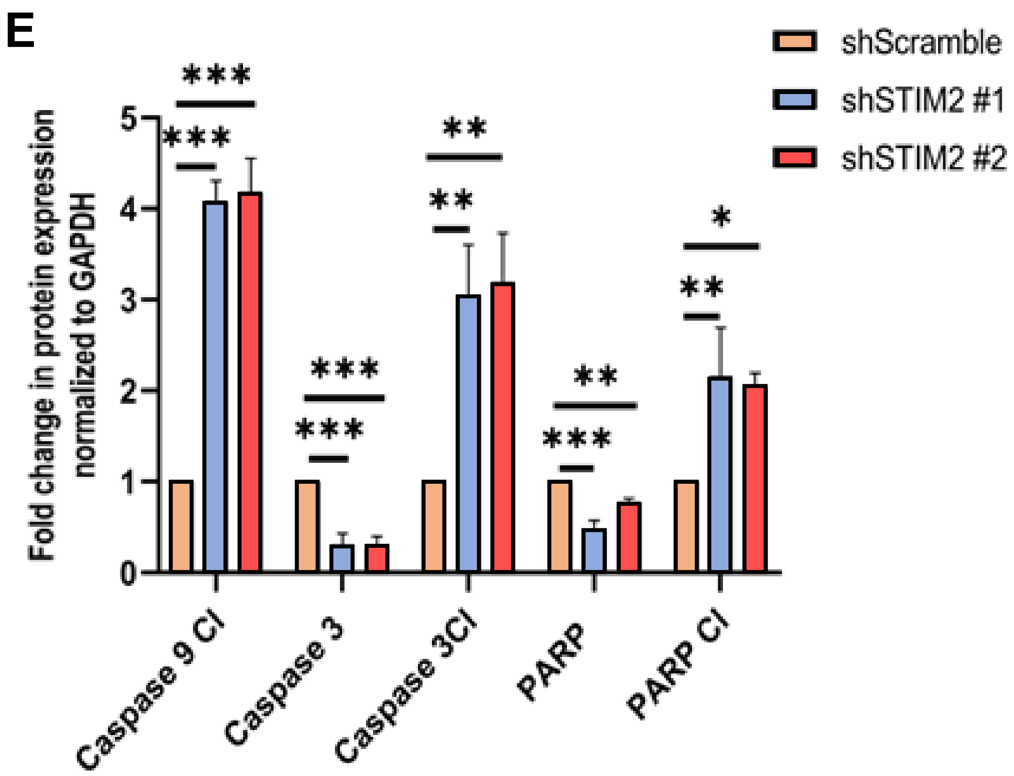

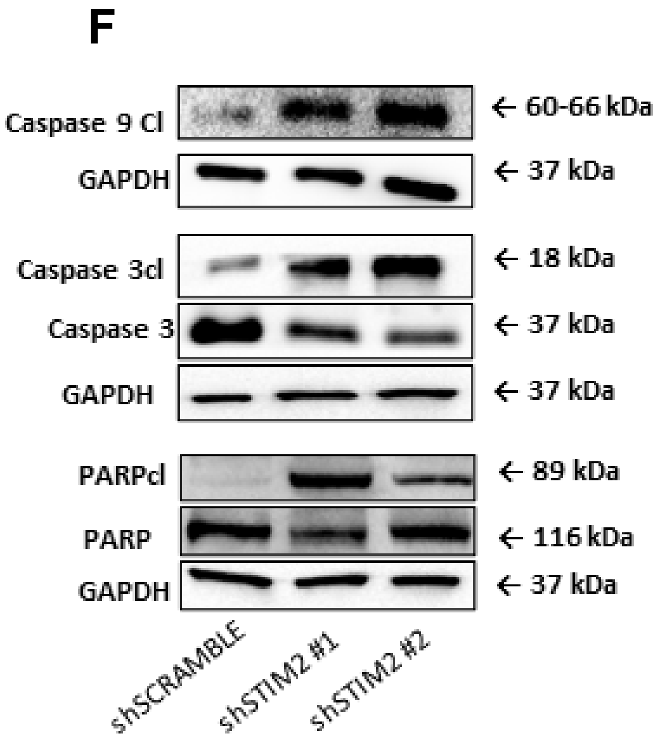

Supplementary Figure 5: Mitochondrial apoptosis in OCI-AML3 cell line. (**A**-**B**) In OCI-AML3, quantification of anti-apoptotic proteins was performed by Western Blot (Bcl-2 - sh#1 : 0,563 ± 0,0115 ; sh#2 : 0,343 ± 0,0929) (Bcl-xL - sh#1 : 0,497 ± 0,0666 ; sh#2 : 0,297 ± 0,0416) (MCL-1 - sh#1 : 0,460 ± 0,147 ; sh#2 : 0,347 ± 0,129) relative to GAPDH and compared to shSCRAMBLE (n=3). (**C**-**D**) Quantification of pro-apoptotic proteins was performed by Western Blot (BAX - sh#1 : 2,60 ± 0,499 ; sh#2 : 2,85 ± 0,805) (BAD - sh#1 : 2,65 ± 0,360 ; sh#2 : 2,32 ± 0,231) relative to GAPDH and compared to shSCRAMBLE (n=3). (**E**-**F**) Membrane and mitochondrial proteins were quantified by Western Blot (Cleaved caspase 9 - sh#1 : 4,08 ± 0,262 ; sh#2 : 4,18 ± 0,373) relative to GAPDH and compared to shSCRAMBLE (n=3). (**E**-**F**) Membrane and mitochondrial proteins were quantified by Western Blot (Cleaved caspase 9 - sh#1 : 4,08 ± 0,262 ; sh#2 : 4,18 ± 0,373) relative to GAPDH and compared to shSCRAMBLE (n=3). (**E**-**F**) Apoptosis effector proteins were quantified by Western Blot (Caspase 3 - sh#1 : 0,307 ± 0,127 ; sh#2 : 0,260 ± 0.137) (Cleaved caspase 3 - sh#1 : 3,05 ± 0,554 ; sh#2 : 3,19 ± 0,538) (PARP - sh#1 : 0,470 ± 0,101 ; sh#2 : 0,773 ± 0.0404) (Cleaved PARP - sh#1 : 2,16 ± 0,534 ; sh#2 : 2,03 ± 0,159) relative to GAPDH and compared to shSCRAMBLE (n=3). ****P<0,001; **P<0,01; *P<0,05*

Supplementary Figure 6

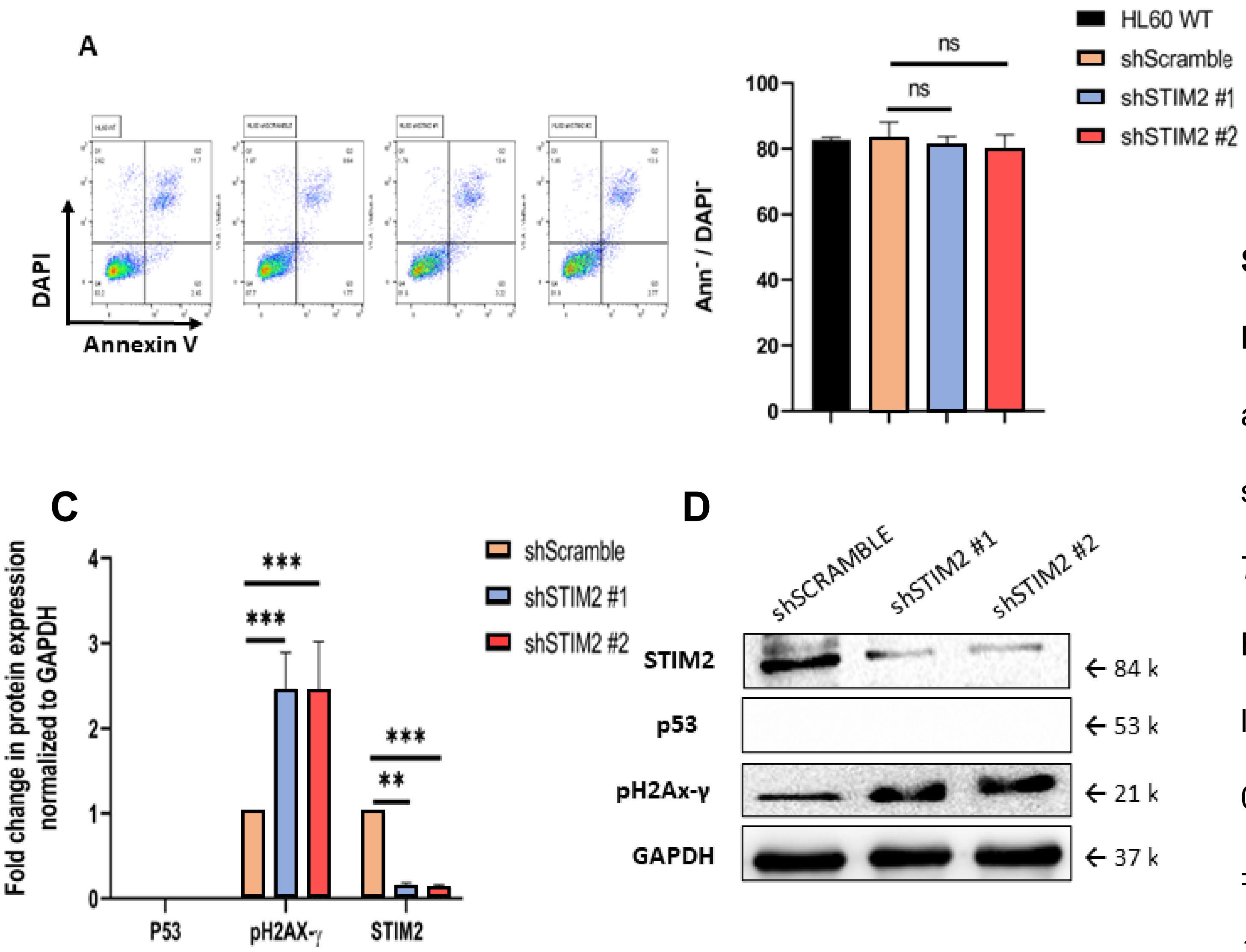

Supplementary Figure 6: Effect of STIM2 knockdown in HL60 cell line. (**A-B**) Cell viability study in HL60 cell line was assessed in flow cytometry (WT : 82,63 ± 0,7371 ; shSCRAMBLE : 83,37 ± 4,805 ; sh#1 : 81,27 ± 2,417 ; sh#2 : 79,9 ± 4,267) (n=3). (**C-D**) p53 null was confirmed by Western Blot (n=3). Quantification of pH2AX-γ and STIM2 at protein level was performed by Western Blot (pH2AX-γ - sh#1 : 2,453 ± 0,4382 ; sh#2 : 2,427 ± 0,5953) (HL60 – STIM2 - sh#1 : 0,1533 ± 0,03055 ; sh#2 : 0,1267 ± 0,03512) (n=3). ****P<0,001; **P<0,01*

Supplementary Figure 7

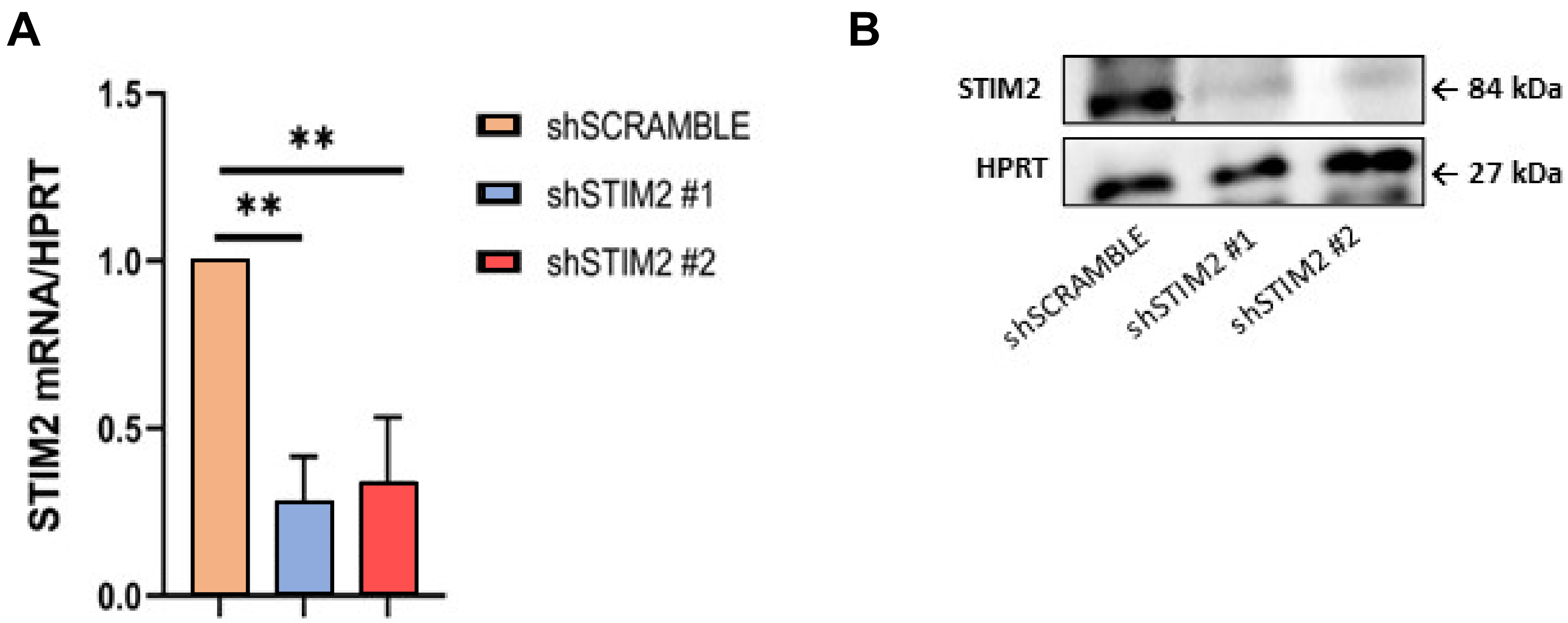

Supplementary Figure 7: STIM2 knockdown in normal hematopoietic stem cell. (**A**-**B**) STIM2 gene expression after shRNA-mediated KD was determined by RT-qPCR (n=3) (sh#1 : 0,283 ± 0,133 ; sh#2 : 0,340 ± 0,193) (**A**) and Western Blot (n=1) (**B**). ***P<0,01*

Supplementary Figure 8

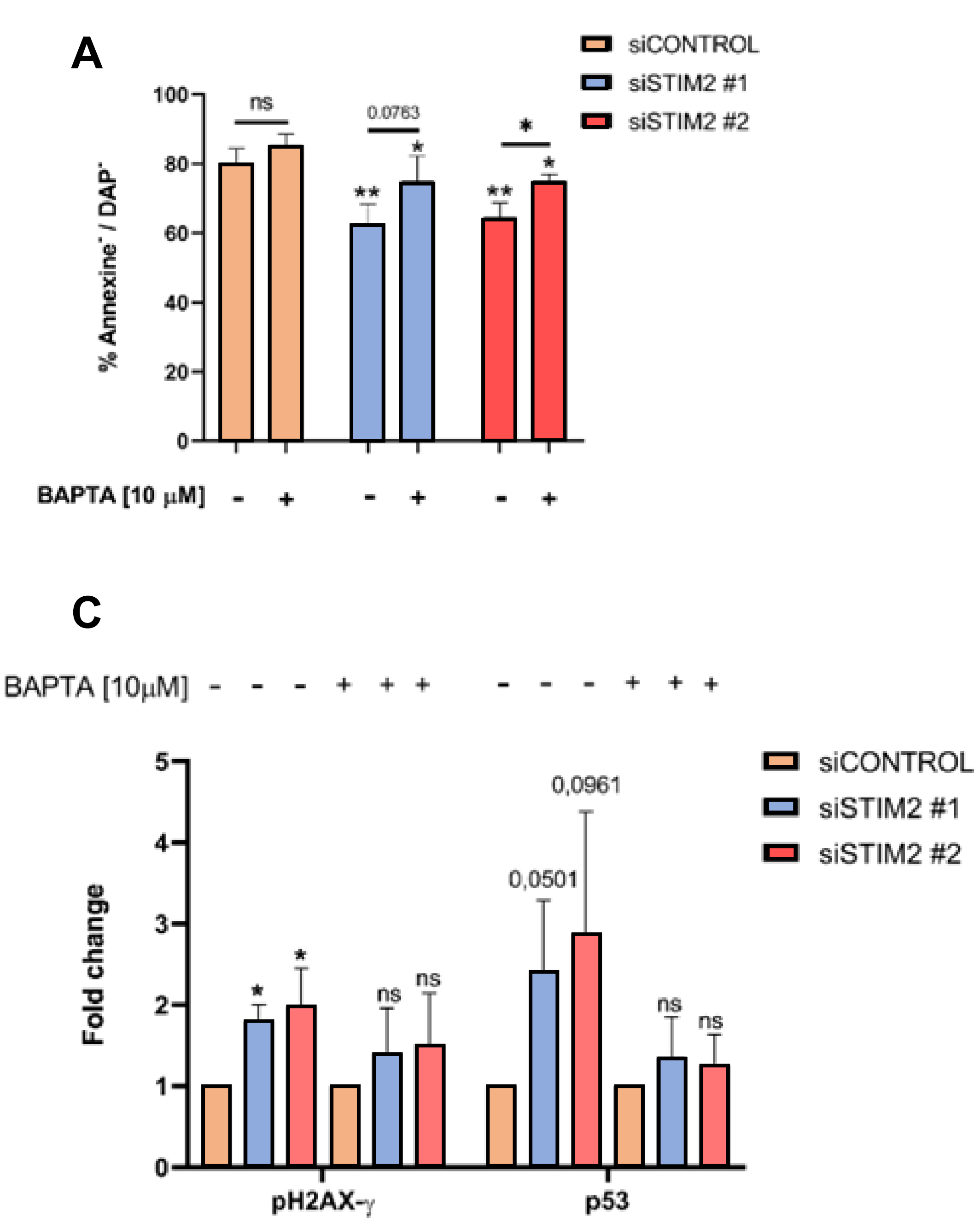

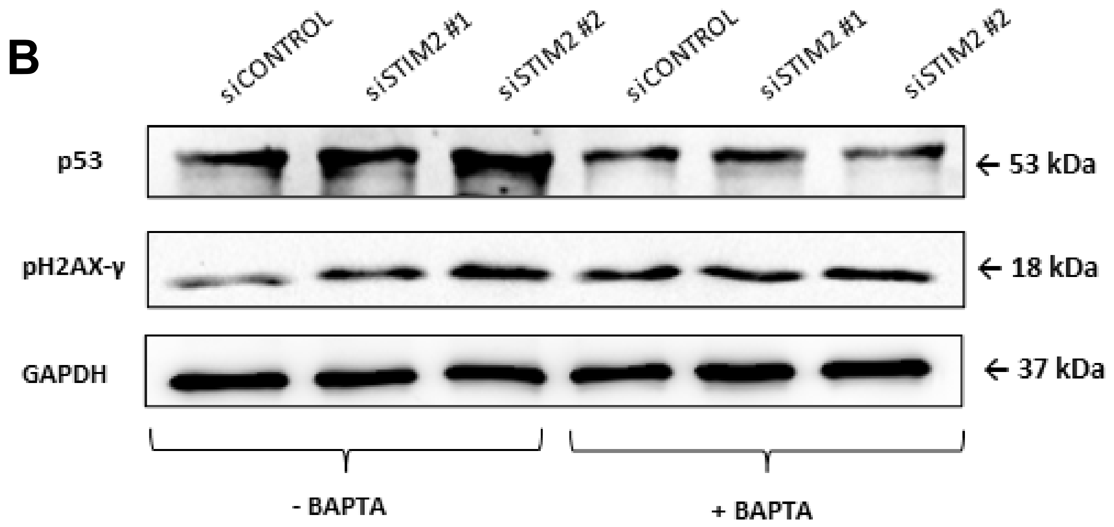

Supplementary Figure 8: BAPTA decrease STIM2 KD-mediated apoptosis in THP-1 cells. (**A**) Cell viability study was assessed in flow cytometry by annexin V/DAPI labeling after STIM2 KD, treated or not with BAPTA (shSCRAMBLE -BAPTA : 80,10 ± 4,215 – shSCRAMBLE +BAPTA : 85,33 ± 3,139 ; si#1 -BAPTA : 62,93 ± 5,383 – si#1 +BAPTA : 75,13 ± 7,071 ; si#2 -BAPTA : 64,63 ± 4,015 – si#2 +BAPTA : 75,00 ± 1,900) and compared to shSCRAMBLE cells (n=3). (**B**) Quantification of p-H2AXγ and p53 at protein level was performed by Western Blot in THP-1 cells after STIM2 KD treated or not with BAPTA (p-H2AXγ - si#1 -BAPTA : 1,797 ± 0,201 – si#1 +BAPTA : 1,410 ± 0,550 ; si#2 -BAPTA : 1,983 ± 0,463 – si#2 +BAPTA : 1,517 ± 0,625) (p53 - si#1 -BAPTA : 2,410 ± 0,880 – si#1 +BAPTA : 2,460 ± 2,571 ; si#2 -BAPTA : 2,880 ± 1,503 – si#2 +BAPTA : 2,160 ± 1,965) (n=3). ***P<0,01; *P<0,05.*
